# Biotechnological advances for improving natural pigment production: a state-of-the-art review

**DOI:** 10.1186/s40643-022-00497-4

**Published:** 2022-01-28

**Authors:** Xiaomei Lyu, Yan Lyu, Hongwei Yu, WeiNing Chen, Lidan Ye, Ruijin Yang

**Affiliations:** 1 School of Food Science and Technology, Jiangnan University, Wuxi, 214122 People’s Republic of China; 2 Institute of Bioengineering, College of Chemical and Biological Engineering, Zhejiang University, Hangzhou, 310027 People’s Republic of China; 3 School of Chemical and Biomedical Engineering, College of Engineering, Nanyang Technological University, Singapore, 637459 Singapore

**Keywords:** Natural pigments, Plant cell/tissue culture, Microbial cultivation, Heterologous biosynthesis, Metabolic engineering

## Abstract

In current years, natural pigments are facing a fast-growing global market due to the increase of people’s awareness of health and the discovery of novel pharmacological effects of various natural pigments, e.g., carotenoids, flavonoids, and curcuminoids. However, the traditional production approaches are source-dependent and generally subject to the low contents of target pigment compounds. In order to scale-up industrial production, many efforts have been devoted to increasing pigment production from natural producers, via development of both in vitro plant cell/tissue culture systems, as well as optimization of microbial cultivation approaches. Moreover, synthetic biology has opened the door for heterologous biosynthesis of pigments via design and re-construction of novel biological modules as well as biological systems in bio-platforms. In this review, the innovative methods and strategies for optimization and engineering of both native and heterologous producers of natural pigments are comprehensively summarized. Current progress in the production of several representative high-value natural pigments is also presented; and the remaining challenges and future perspectives are discussed.

**Graphical Abstract:**

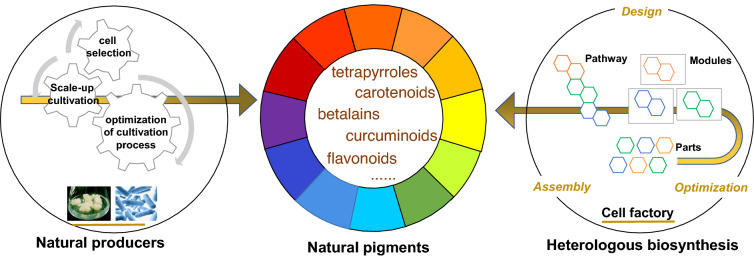

## Introduction

Pigments are defined as finely divided and usually water-insoluble colorants, absorbing and reflecting the visible light to show different colors (Rapp 2009). They are of larger molecular weight, less water-soluble, and less transparent than dyes. The usage of pigments has a long history, dating back to the beginning of ancient civilizations (ancient China, India, and Egypt), whereby natural plants, insects, and minerals were used to dye textiles, color foods, paint, color the body in religious ceremonies, and more. In 1856, English chemist William Henry Perkin discovered the first synthesized organic dye from coal tar distillate (Chandler 2001), which triggered the commercial flourishing of synthetic pigments. However, they are derived from chemical reactions, causing heavy environment stress. More notably, many serious health hazards such as toxicity, oncogenicity, and teratogenicity were found associated with synthetic pigments (Li and Tian 2017).

With the increase in public health awareness, replacement of synthetic pigments with safer and more ecofriendly natural pigments has become the current marketing trend. According to the report by GRAND VIEW RESEARCH, the global market for natural pigments in food industry alone is estimated to reach 2.5 billion USD by 2025 (https://www.grandviewresearch.com/industry-analysis/food-colorants-market). Besides usage as colorants, many of natural pigments show great potential in pharmaceutical, nutrition, and skincare industries, due to their health benefits (Amalraj et al. 2017; Chen and Zhong 2015; Yamagata et al. 2015). For instance, as one of the most bioactive xanthophyll pigments that mainly presents in higher plants and microalgae, lutein can prevent and treat retinal damage, including age-related macular degeneration, glaucoma and diabetic retinopathy (Krinsky et al. 2003; Seddon et al. 1994; Sun et al. 2011; Zhang et al. 2016a). It has also been shown to prevent cardiovascular diseases, atherosclerosis and cancers (Astorg 1997; James et al. 2001). The global market for lutein is fast growing, which is expected to reach 357.7 million USD and 2121.2 tons by 2022 (Markets 2017).

According to their structural characteristics, natural pigments can be classified into tetrapyrroles, carotenoids, flavonoids, curcuminoids, betalains, quinones, and others. They are widely distributed across all the natural kingdoms, e.g., plants, animals, and microorganisms. However, each natural source has its own limitations and currently cannot compete economically with synthetic counterparts. Taking astaxanthin (a typical carotenoid compound) as an example, its extraction from green microalga is subject to the lengthy autotrophic cultivation in open freshwater ponds (10–15 days) and the requirement of cell wall disruption to release the carotenoids (Mota et al. 2021); the frequently reported astaxanthin contents from crustaceans, salmon, and trout sources are less than 1 mg/g (Ahmadkelayeh and Hawboldt 2020); the yield of astaxanthin in most wild strains of the microorganism *Phaffia rhodozyma* can only reach 0.3–0.9% while in bioreactor high cell density and production titers can be achieved in much shorter times (Wan et al. 2021). Therefore, improving biotechnological productivity towards achieving large-scale production is presently an important challenge.

To date, great efforts have been devoted to in vitro production of natural pigments via plant cell/tissue culture and optimization of microbial cultivation. Moreover, rational engineering via pathway mining and genetic engineering has been proposed and applied to improve the yield of various natural pigments. Although considerable achievements have been obtained from biotechnological modification of natural producers, in most cases their productivity still cannot meet the requirements for industrial-scale production.

At the beginning of the twenty-first century, the emergence of “omics” techniques greatly improved our understanding of natural biological systems. Based on these knowledge treasures, synthetic biology came into the forefront, whereby engineering principles were employed to design and reconstruct novel biological modules as well as biological systems (Osbourn et al. 2012). By constructing artificial metabolic pathways using designed parts in well-known biological systems, unlimited supply of previously expensive or unfeasible products could be realized. Currently, model microorganisms like *Escherichia coli* and *Saccharomyces cerevisiae* are regarded as the ideal chassis for engineering, owing to their genetic tractability, short cycle of life, and mature technology of high-density fermentation. One classic example is the production of crocins (Fangyu et al. 2018; Liu et al. 2020), which demonstrates the potential of synthetic biology in realizing large-scale production of rare and high-value natural pigments. The simultaneous blooming of genome sequencing, genetic editing, and computer modeling techniques further accelerated progress in heterologous biosynthesis, ushering in the dawn of industrial production of natural pigments.

During the past two decades, several reviews (Begum et al. 2016; Lagashetti et al. 2019; Manivasagan et al. 2018; Rodriguez-Amaya 2016; Sigurdson et al. 2017) have been published on the topic of natural pigments. These articles mainly focused on the characteristics, biosynthesis, regulation, functions and applications of pigments, while comparatively little information on the biotechnological progresses in their production was given. More recently, certain pigments (mainly carotenoids) have been well reviewed, covering their potential use, chemistry and biosynthesis, economic importance, and strategies to enhance production (Heider et al. 2014; Hu et al. 2018; Mussagy et al. 2019b; Saini et al. 2020; Saini and Keum 2019; Venil et al. 2013; Wang et al. 2021a). However, readers cannot get a full picture of the progresses and challenges in industrial application of natural pigments due to the limited categories of pigments and producers (like microalgae) covered in those reviews. Furthermore, the biotechnological engineering methods and strategies are rarely systematically analyzed and summarized. In this review, we present a systematic introduction on the progresses in biosynthesis of natural pigments with the aim to provide a more complete picture of natural pigments development. Specifically, part 2 introduces the current progresses in improving pigment production from natural producers; part 3 summarizes the technical developments towards efficient heterologous biosynthesis of pigments in non-pigment producing organisms; part 4 focuses on representative high-value products among natural pigments, and comprehensively reviews the efforts made in promoting their biosynthesis; lastly, part 5 discusses the challenges and future perspectives in industrial application of natural pigments.

## Biosynthesis of pigments from natural producers

### Plant cell culture and tissue culture

**Fig. 1:**
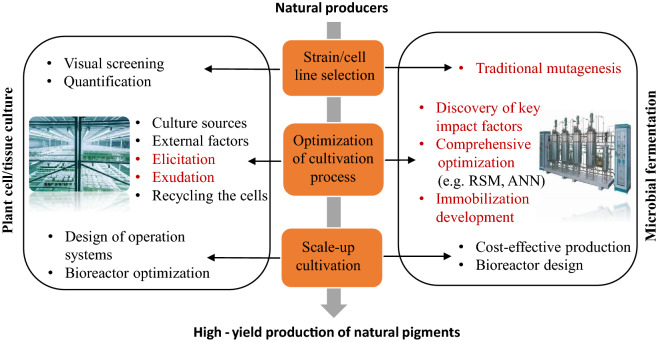
Routes and strategies for improving production of pigments from natural producers, by optimization of traditional methods including plant cell/tissue culture (left) and microbial cultivation (right)

Plants are the most abundant source of natural pigments, including chlorophyll, anthocyanin, carotenoids, betalains, etc. Traditionally, most of the commercial pigments are derived from natural sources, either foraging or large-scale plant field cultivation. Due to the low accumulated contents of desired biochemicals in the whole plant, this approach leads to heavy consumption of farmlands and damage to precious natural plants. Taking crocetin—the most important active ingredient and pigment mainly distributed in the stigmas of *Crocus sativus*, as an example, it is estimated that 20 g of crocetin (in 1 kg of dry *Crocus sativus* stigma) requires 110,000–170,000 flowers and over 400 h of hand-labor (Frusciante et al. 2014). Besides, *Crocus sativus* grows slowly and only propagates by vegetative production. Plant cell culture is a technique which can enable plant cells to produce the full spectrum of characteristic second metabolites as mother plants under strictly controlled environmental conditions (Georgiev et al. 2018). In vitro plant production approach is independent of geographical and seasonal limitations, providing an alternative approach to increase the productivity of plant metabolites of interests. Selection and development of high-yielding cell lines as well as optimization of cell cultivation process, including optimization of culture conditions, recycling the cells via immobilization, and bioreactor redesign for scale-up cultivation, are the most commonly used strategies for improving metabolite accumulation (Fig. 1) (Bhojwani and Dantu 2013). As these traditional strategies in pigment production have been summarized in previous reviews (Murthy et al. 2014; Steingroewer et al. 2013), herein, we will mainly focus on two extension techniques for triggering the biosynthesis of secondary metabolites based on bionic innovation (elicitation) and continuous release of desired bioactive compounds (exudation).

#### Elicitation

The biosynthesis of secondary metabolites in plants is activated in response to pathogens (or insects) attack or various abiotic stresses (e.g., radiation, heavy metal and mineral, temperature, salinity) (Ramirez-Estrada et al. 2016). These stress signaling compounds are designated as “elicitors”, the majority of which are perceived by membrane receptors first and entail various sequential reactions (including phosphorylation or dephosphorylation of plasma membrane proteins, [Ca^2+^] enhancement, Cl^−^and K^+^ efflux/H + influx, NADPH oxidase activation, defense gene expression, and secondary metabolite biosynthesis) to trigger the biosynthesis of secondary metabolites (Ramirez-Estrada et al. 2016; Zhang et al. 2012). According to the studies on production of the pigments—betalain, shikonins, carotenoids and flavonoids (Georgiev et al. 2008; Malik et al. 2016; Rizzello et al. 2014; Savitha et al. 2006), biotic elicitors can be divided to four classes: (1) exogenous compounds secreted by microorganisms and insects when attacking plants (Warhade and Badere 2018); (2) exogenous compounds formed from the degradation of microbial cells by plant enzymes, such as fungal and bacterial lysates and polysaccharides of the microbial cell walls (e.g., chitin, glucans) (Bahabadi et al. 2014; Tang et al. 2019); (3) plant cell wall fragments degraded by pathogens; (4) the intracellular proteins or compounds synthesized by plants in response to pathogen attack or abiotic stress (e.g., plant hormones). On the other hand, many abiotic elicitors (like heavy metals, UV irradiation, and inorganic salts) also play important roles in activating plant secondary product biosynthesis.

#### Exudation

Plant pigments are usually stored in different tissues, making their isolation difficult. A considerable number of studies have been executed to export pigment compounds, which can be summarized into three methods: (1) membrane permeabilization using chemical agents (e.g., Tween 20, DMSO, isopropanol, Triton X-100, polysaccharides like chitosan, and high ionic strength) or physical approaches (e.g., ultrasonication, pulsed electric fields, and high hydrostatic pressure) (Saw et al. 2012); (2) in situ extraction (e.g., usage of *n*-hexadecane and liquid paraffin in liquid–liquid system) (Zare et al. 2010); (3) introduction of specific transport mechanisms, including vesicle trafficking, membrane transporters, and glutathione S-transferase (GST) (Zhao 2015). However, the specific transporters as well as the mechanisms are still poorly understood, which may be a future research focus.

### Microbial cultivation

#### Traditional mutagenesis

Typical pigment-producing microorganisms include *Phaffia rhodozyma* (yeast, producing red pigments—carotenoids), *Monascus* sp (fungus, producing monascus red pigments), *Blakeslea trispora* (fungus, producing orange pigments like beta-carotene), *Streptomyces cyaneus* (fungus, producing black pigment—melanin), and *Serratia *sp (bacteria, producing red pigment—prodigiosin) (Chao et al. 2018; El-Batal et al. 2017; Kim and Ku 2018; Mussagy et al. 2021; Sun et al. 2021). Traditional random mutagenesis using ultraviolet (UV), 1-methyl-3-nitro-1-nitrosoguanidine (NTG) and ethyl methane sulfonate (EMS) have greatly improved microbial pigment production (Yolmeh and Khomeiri 2016; Yolmeh et al. 2017). Nevertheless, these methods suffer from severe drawbacks such as long searching time and low success rate.

#### Discovery of key impact factors and comprehensive optimization

The accumulation of pigments is generally related to cell growth and affected by nutrient factors (carbon source, nitrogen source, C/N ratio), microbiological parameters (spores age, seed age, inoculum age) and environmental conditions. In terms of environmental conditions, several essential aspects should be evaluated and optimized for the solid-state fermentation (SSF)—the processes performed on non-soluble materials. These aspects include the humidity of medium, physical and structural properties of substrates, temperature, pH and agitation (de Castro and Sato 2015). The low moisture content indicates that this cultivation process can only be carried out by a limited number of microorganisms, mainly yeasts and fungi. The other cultivation process is submerged fermentation (SmF) which based on liquid culture for most microorganisms, and is highly influenced by factors including temperature, pH, and agitation (Morales-Oyervides et al. 2020). Furthermore, it was revealed that production of some pigments is induced by certain stress conditions. For instance, Velmurugan et al. demonstrated that total darkness stimulated accumulation of pigments in *Monascus purpureus*, *Isaria farinosa*, *Emericella nidulans*, *Fusarium verticillioides* and *Penicillium purpurogenum* (Velmurugan et al. 2010). For photosynthetic microorganisms, light sources, light intensity, and light photoperiod were illustrated to be the key factors affecting pigment production (Cheirsilp and Torpee 2012; Kuo et al. 2012; Liu et al. 2019b; Zhou et al. 2015b).

Based on discovery of the key impact factors, comprehensive optimization is required to obtain high-yield production of desired pigments. Response surface method (RSM) can solve multivariate data from appropriately designed experiments, and help improve pigment production by optimization of culture medium (Seyedin et al. 2015), process parameters (Sehrawat et al. 2017), and extraction conditions (Zhong et al. 2019). The recently emerged artificial neural networks (ANN) is more flexible and accurate in comparison to RSM (Shafi et al. 2018), having proved effective in improving the production of red pigment in *M. purpureus* MTCC 369 (Singh et al. 2015).

#### Development and application of novel immobilization methods

Immobilization technology provides a feasible approach to improve the stability and reusability of the cells, facilitating easier downstream cell separation and continuous operation. Immobilization of bacteria and yeast has been applied for production of carotenoids (Alipour et al. 2017). For molds, SSF is deemed to be superior to liquid-state fermentation (LSF) in pigment production. Extensive studies showed that immobilization cultivation by using cell entrapment and adsorption methods can mimic the solid-state environment of SSF and effectively improve pigment production from molds (Liu et al. 2010). To eliminate nutrient mass transfer limitation from traditional immobilization, membrane-surface liquid culture (MSLC) and modified system-self-immobilization biomembrane-surface liquid culture were developed, whereby the molds could grow on the upper side of the membrane and the other side contacts with liquid medium for supplementation of nutrition (Wang et al. 2012). On the other hand, cultivation of phototrophic dinoflagellates, which can produce pigments like carotenoids and xanthophyll peridinin, is hindered by their sensitivity to hydrodynamic stress. Immobilization of microalgae in a biofilm on sheet-like surfaces could circumvent this problem (Olivieri et al. 2014). Employment of biofilm photobioreactors for cultivation of marine dinoflagellate *Symbiodinium* led to significantly higher biomass yield and peridinin productivity over suspension culture (Benstein et al. 2014).

### Rational engineering of natural producers

#### Mining of natural pathways

##### Pre-omics

**Fig. 2:**
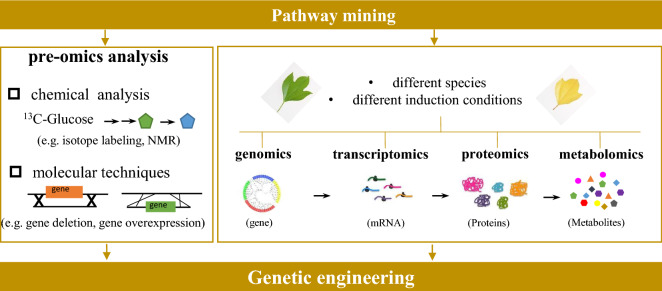
Overview of strategies for exploration of metabolic pathways in nature producers. Pre-omics analysis methods consist of chemical analysis (e.g., isotope labeling, NMR analysis) and molecular techniques (e.g., gene deletion, gene expression, DNA sequencing by genome walking); omics technologies include genomics, transcriptomics, proteomics and metabolomics. Both of them offer powerful tools to discover new genes, gene clusters, transcription factors as well as pathways for synthesis of bioactive compounds

Isotope labeling (^1^H, ^3^H, ^13^C and ^15^ N) and nuclear magnetic resonance spectroscopy (NMR) analyses can give clues on the biosynthetic pathways via direct observation of isotopic shifts. In the study by Dong et al. (2021), a dual stable isotope labeling approach named DLEMMA was employed to identify and track phenylpropanoid pathway in Arabidopsis thaliana. It was found that PAP1 was a key enzyme for accumulation of cyanidin-type anthocyanins and quercetin-type flavonols. In addition to chemical analysis, a variety of molecular techniques have been employed to identify genes and gene clusters responsible for production of the target metabolites (Fig. 2). The most common strategies can be summarized as: (1) gene deletion and complementation (Bitok et al. 2017); (2) amplification of target gene based on conserved sequences of known enzymes from other organisms and other methods derived thereof (Jones et al. 2008); (3) sequencing the DNA fragments flanking both sides of the known functional enzymes by genome walking (Shapter and Waters 2014); and (4) using a known gene as the bait to reveal unknown regulators or related genes (Lu et al. 2018).

##### Omics

In recent years, combined applications of “omics” techniques—via comparative systematic analysis among different species or the same species under different cultivation/induction conditions—offer powerful tools to discover new genes, gene clusters, transcription factors as well as pathways for synthesis of bioactive compounds (Fig. 2). Via transcriptome analysis of purplish-red leaf and green leaf of Paeonia qiui, several new candidate genes influencing anthocyanin accumulation were discovered, including MYB1, MYB2, bHLH1 and WD40-1 (Luo et al. 2017). Integrated transcriptomics and metabolomics in tomato revealed 38 candidate carotenoid-correlated genes and demonstrated the important role of the ethylene response factor *SIERF6* in ripening and carotenoid production (Lee et al. 2012).

Omics-based information on gene distribution and expression regulation can also be used to guide the discovery of unknown genes or clusters (Medema and Osbourn 2016). As demonstrated by Shimura et al., new metabolic pathways or unknown genes can be discovered based on the identification of physically clustered groups (Shimura et al. 2007). Another typical rule is “co-expression”: specialized metabolites are synthesized in certain cell types, growth phases, or in response to environmental induction, for which the corresponding genes usually co-ordinate transcripts activated by specific transcription factors. Therefore, if the gene encoding one of the pathway enzymes has been identified, the other functional enzymes in the same pathway might be elucidated by co-expression analysis using transcriptomics and by metabolites analysis via metabolomics (Geu-Flores et al. 2012).

#### Genetic engineering of natural producers

The rapid development of bioinformatics, sequencing technology, and genetic engineering has enabled gene manipulation of natural producers to improve pigment production. Herein, we take the progress of *Monascus* azaphilone pigments (MonAzPs) as an example for illustration. MonAzPs have been used as food colorants and food preservatives in East Asian countries for more than a millennium, while the genetic background of *Monascus sp.* for producing red pigments remained unclear until recently. In 2013, the key gene cluster (the PKS-FAS gene cluster) involved in the biosynthesis of azaphilone pigments was identified by T-DNA random mutagenesis in *M. purpureus* (Balakrishnan et al. 2013), whereby MppR1 and MpPKS5 were confirmed as the key enzymes. The complete genome information of *M. purpureus* YY-1 was obtained by next-generation sequencing and optical mapping techniques, which together with transcriptomic analyses revealed the potential biosynthesis pathway and the regulatory mechanism of pigment production (Yang et al. 2015). On this basis, the individual steps of MonAzPs pathway of *M. ruber* M7 were elucidated by a systematic functional investigation (Chen et al. 2017b). These advances in elucidating genetic background of natural pigment producers give a roadmap towards improved synthesis of desired products. Downregulation of the citrinin biosynthesis, a competing metabolic branch to MonAzPs biosynthesis, improved the production of pigments by 60% (Liang et al. 2018). Disruption of the ergosterol biosynthetic pathway increased membrane permeability and led to the secretion of *Monascus* pigments, resulting in 2.06-fold increase in pigment production (Liu et al. 2019a). In short, upregulation of the MonAzPs biosynthetic pathway, complemented with downregulation of competing metabolic branches and improvement of product secretion, offers effective approaches for enhancing *Monascus* pigments in the native producers. Apart from MonAzPs, genetic engineering is being performed in natural producers of other pigments, such as carotenoids (Ganapathy et al. 2016). For further improvement, the difficulty lies in the lack of understanding on the regulation of pigment synthesis.

## Efforts towards heterologous biosynthesis of pigments

Despite the great progress achieved thus far in pigment production by natural biosynthesis, the yield of target compounds remains limited due to low productivity and incomplete understanding of the genetic background of natural producers. Over the past two decades, metabolic engineering has emerged as a powerful tool to develop heterologous cell factories for producing natural pigments. Herein, the efforts towards heterologous biosynthesis of pigments, in the aspects of pathway design, pathway construction and pathway optimization are summarized.

### Pathway design

The first critical step for heterologous biosynthesis of pigments is the identification of functional genes and responsible pathways. Natural synthetic pathways usually suffer from metabolic bottlenecks caused by the presence of rate-limiting enzymes and feedback inhibition. Synthetic biology provides an approach to design novel pathways via assembly of catalytic elements and pathway modules from diverse organisms so as to circumvent low-activity enzymes and avoid feedback regulation. For example, insufficient isopentenyl diphosphate (IPP) and dimethylallyl diphosphate (DMAPP) supply is a common bottleneck for accumulation of carotenoids. Introduction of a recombinant mevalonate pathway (MVA pathway) consisting of the bottom portion from *Streptococcus pneumonia* and the top portion from *Enterococcus faecalis* led to increased β-carotene production in *E. coli* (Yoon et al. 2009). For production of anthocyanins and flavonoids, shikimate pathway (SK) is crucial for providing the key precursor coumaric acid. In SK, the conversion of 2-dehydro-3-deoxyarabinoheptulosonate-7-phosphate (DAHP) into the important intermediate EP3P was catalyzed by only Aro1 in yeast systems. In contrast, this catalysis requires the cooperation of seven distinct enzymes—AroB, AroD, AroE, YdiB, AroL, AroK, and AroA, in *E. coli*. The addition of aroL from *E. coli* into the native SK in yeast displayed a positive effect on *p*-coumaric acid production (Rodriguez et al. 2015).

### Pathway construction

**Fig. 3:**
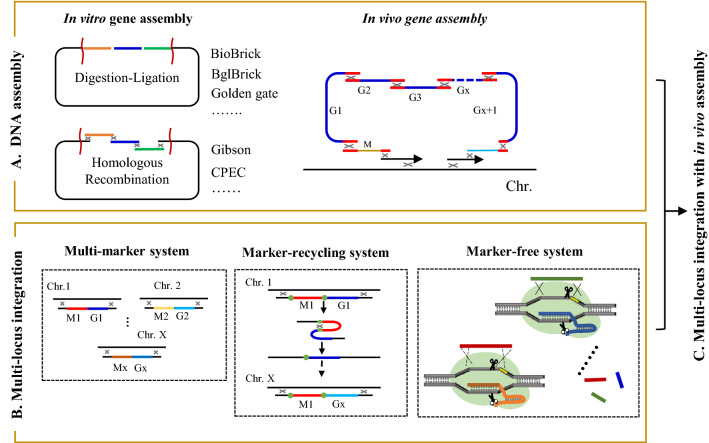
Overview of innovative methods for pathway assembly. **A** Assembly of multiple genes into one large plasmid or one genomic locus. Restriction digestion/ligation-based methods with usage of type II and type IIs restriction enzymes are employed for in vitro gene assembly. Sequence homology-based methods are adopted to join multiple DNA fragments within one step in vivo or in vitro via short overlap. **B** Multi-locus integration of long pathway by splitting the whole pathway into several segments and then inserting them into multiple sites within one step or by multi-round integration, utilizing multiple markers, recycling markers or adopting marker-free systems. **C** Combination of multi-locus integration approach and in vivo assembly method to facilitate long-pathway construction

The long biosynthetic pathway of complex natural pigments, such as carotenoids, flavonoids and quinones, raises the issue of pathway assembly in heterologous hosts. Construction of multiple plasmids using the traditional approach involving multiple cloning steps such as PCR, endonuclease digestion and ligation, is time-consuming, inefficient, and limited by the availability of restriction sites in the plasmids. In recent years, novel DNA assembly methods and multi-locus integration methods have been developed and adopted for pigment production (Fig. 3).

#### DNA assembly

Restriction digestion/ligation-based methods using type II and type IIs restriction enzymes such as BioBrick (Shetty et al. 2008), BglBrick (Lee et al. 2011) and Golden gate (Engler et al. 2009), are the typical in vitro gene assembly method for large plasmid construction. The other type of in vitro gene assembly method is the sequence homology-based methods like Gibson (Gibson et al. 2009) and CPEC (Quan and Tian 2009), whereby multiple DNA fragments can be easily joined together within one step via short overlaps (Fig. 3A). Apart from in vitro gene assembly methods, in vivo HR-based methods like *E. coli* RecET/Red system (Fu et al. 2012) and *B. subtilis* DNA assembly (Itaya et al. 2008) have been developed and employed for pathway construction and even chromosome creation. In in vivo DNA assembler, each DNA cassette is designed with > 40 bp homologous arms. Based on simple operation plus the high efficiency of the native HR mechanism within yeast, several DNA fragments could be assembled and inserted into the designated sites in the chromosome. By employment of an in vivo DNA assembler, a xylose-utilizing zeaxanthin biosynthetic pathway consisting of eight genes (~ 19 kb) was assembled within one-step transformation in yeast (Shao et al. 2009). As compared to in vitro gene assembly, the in vivo gene assembly via employment of the intracellular HR mechanism may omit the addition of cloning enzymes which may lower cloning efficiencies.

#### Multi-locus integration

Although DNA assembly provides an effective approach for rapid construction of long pathways, successful rate could be affected depending on whether all bioparts are assembled in a single designated site. As an alternative strategy, multi-locus integration splits the pathway into several segments and inserts them into multiple sites via one-step manipulation or multi-round integration (Fig. 3B). The conventional way is to integrate target genes in different loci using different markers, but this strategy is limited by the low available number of selection markers. Cre-loxp-based system and URA-FOA negative selection were then developed for removal and reuse of selection marker (Aguiar et al. 2014). Inspired by these designs, we developed a marker recyclable integrative toolbox for pathway assembly in *S. cerevisiae*, with the “loxp-kanMX-loxp” cassette conferring resistance against the antibiotic G418 and meanwhile facilitating marker recycling (Ye et al. 2017)*.* By combining this with the decentralized assembly strategy, a total of 11 genes encoding the carotenoid biosynthetic pathway, with length of ~ 22 kb, was integrated into the genome of yeast within 5 rounds of reiterative recombination, leading to successful heterologous production of 16.3 mg∙g^−1^ dcw carotenoids.

In order to improve the efficiency of HR and achieve marker-free genomic engineering in the multi-locus integration method, several CRISPR–Cas9-facilitated multiplexed integration strategies, such as delta integration CRISPR–Cas (Shi et al. 2016), “Wicket” (Hou et al. 2018), co-transformation of multiple gRNA and donor DNA cassettes (Horwitz et al. 2015), have been developed for assembly of long pathways or modulating the copy numbers of integrated genes. Taking “wicket” design as an example, a short DNA harboring two 50 bp HAs and a 23 bp CRISPR–Cas9 target sequence were designed as docks for integration of exogenous DNA fragments (Hou et al. 2018). Using this approach, multicopies of β-carotene synthetic genes *crtE*, *crtYB* and *crtI* were integrated into the yeast genome, without needing any selective marker.

#### Multi-locus integration with in vivo assembly

As mentioned previously, both DNA assembly and multi-locus integration can significantly facilitate long-pathway construction. In 2015, the Jay Keasling group developed a new method, named CasEMBLR, for multi-DNA assembly via a combination of multi-locus genomic integration method and in vivo DNA assembly with the usage of CRISPR/Cas9 system. To validate its applicability, 15 DNA parts for carotenoid biosynthesis were successfully assembled and integrated into three targeted genomic loci (*URA3, HIS3* and *ADE2*) of yeast (Jakociunas et al. 2015).

### Pathway optimization

**Fig. 4:**
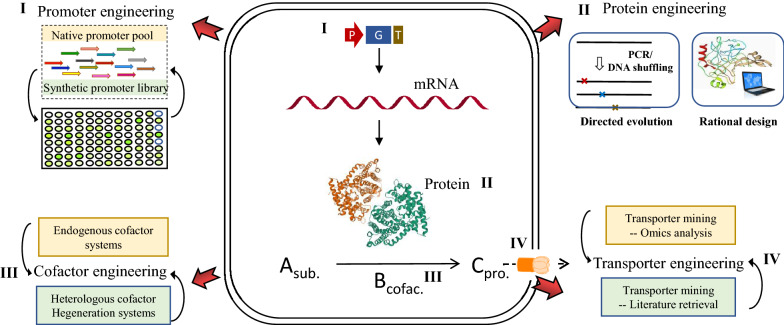
Overview of strategies for engineering of rate-limiting bio-parts in heterologous biosynthesis. **I** Promoter engineering is used for fine-tuning of gene expression via screening and characterization of native promoter pool as well as construction of synthetic promoter library. **II** Protein engineering is employed to optimize enzymatic properties via directed evolution and rational design.** III** Cofactor engineering is adopted to improve the supply of reducing forces by regulation of endogenous cofactor system or introduction of heterologous cofactor regeneration systems. **IV** Transporter mining is applied to eliminate the transportation bottlenecks via omics analysis and literature retrieval

**Fig. 5:**
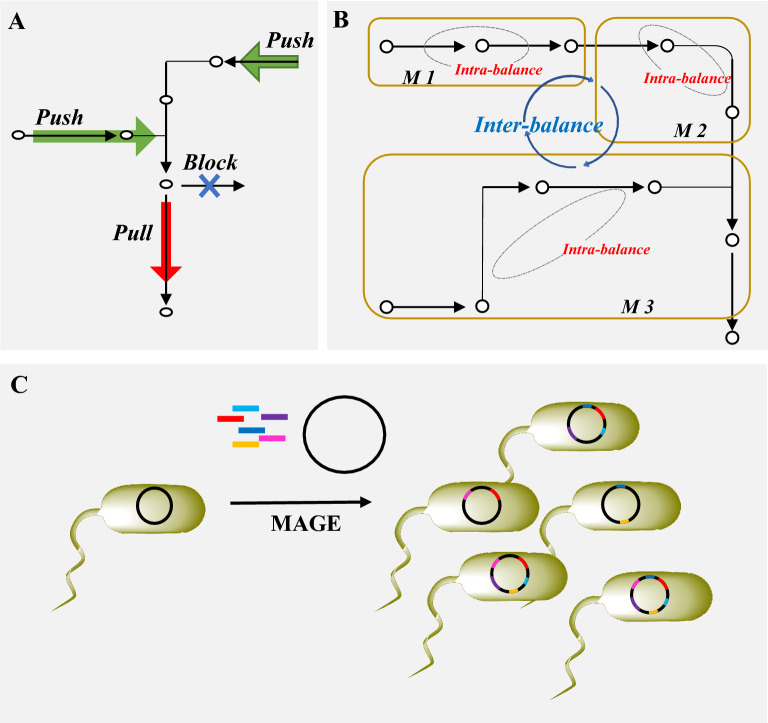
Overview of strategies for engineering of metabolic network in heterologous biosynthesis. **A** “Push–pull–block” strategy is used to direct more metabolic flux to the target compounds, in which “push” indicates enhancing the precursor supply towards target products, “pull” means strengthening the formation ability of the target products, and “block” represents downregulation of the competing pathways. **B** Inter- and intra-modular pathway engineering strategy is proposed to regulate metabolic balance among multiple modules as well as individual genes within each module. **C** Multiplex genome engineering like multiplex automated genome engineering (MAGE) is applied for large-scale programming of cell factory. In MAGE, synthetic ssDNA pools (color lines) are transformed into engineered hosts for generation of a diverse set of genomic modifications

**Fig. 6:**
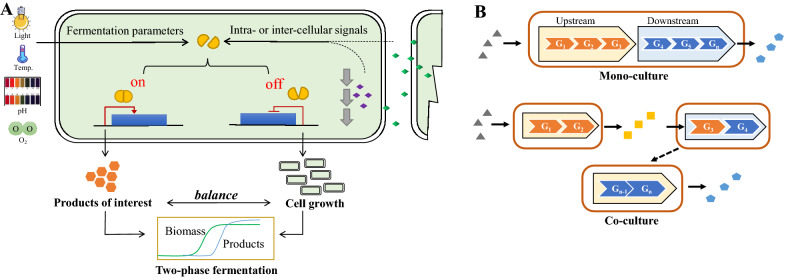
Overview of strategies for engineering of cellular systems in heterologous biosynthesis. **A** Two-stage cultivation is employed to maintain normal cell growth while maximizing pigment production, in which environmental signals (like temperature, light, pH and oxygen) and intra-/inter-cellular signals (like QS system) are used as inducers to trigger the switch-on and switch-off of gene expression. **B** Co-culture engineering provides an alternative approach for efficient pathway engineering via dividing the complex long pathways into several pathway sections in different strains

Introduction of a heterologous pathway can endow the cell factory with the capacity to produce desired compounds, however the productivity is generally low due to the complexity of metabolic networks in all living systems. For instance, rate-limiting steps may result from undesirable biological characteristics of bio-parts (e.g., promoter, enzyme, cofactor, transporter); metabolic flux to the target compound may be limited by feedback regulation, diverse competing branches or insufficient precursor supply; moreover, overexpression of proteins may lead to heavy metabolic burden, resulting in significant biomass decrease. Therefore, pathway optimization is essential to overcome these bottlenecks for achieving high-efficiency bioproduction. Herein, pathway optimization strategies and their applications in pigment biosynthesis are illustrated, classified into three levels: engineering of rate-limiting bio-parts/factors (“Engineering of rate-limiting bio-parts/factors” Section , Fig. 4), engineering of the metabolic network (“Engineering of the metabolic network” Section, Fig. 5), and engineering of cellular systems (“Engineering of cellular systems” Section, Fig. 6).

#### Engineering of rate-limiting bio-parts/factors

##### Promoter engineering

Precise regulation of gene expression is critical to improve product formation and cell growth, as excessive gene expression will generate metabolic burden while insufficient expression may cause accumulation of metabolic intermediates. Promoter optimization is the most direct way to modulate gene expression via controlling the transcription level of specific genes. However, its application is dependent on the availability of endogenous promoters with proper feature. To enrich promoter sources, the native promoter pools in microorganisms and plants are screened via omics and characterized using reporters, and synthetic promoter libraries have been constructed by means of error-prone PCR, site-directed mutagenesis, saturation mutagenesis, and hybrid-promoter design (Fig. 4I). As these above methods have been previously reviewed (Xu et al. 2019; Zhou et al. 2017c), they shall not be described in detail here.

##### Protein engineering

Enzymes are the basic catalytic elements for biosynthesis of natural products. Innate enzymatic properties, including catalytic activity, substrate specificity and allosteric regulation characteristics, are often incompatible with the engineering hosts. Poor performance of enzymes may generate bottlenecks, unwanted by-products and feedback inhibition, resulting in low synthetic efficiency and productivity.

Directed evolution can mimic Darwinian evolution to achieve functional changes by altering the enzyme structure, and the process typically comprises library construction and artificial selection (Fig. 4II). Random mutations can be introduced into the protein via either error-prone PCR or DNA shuffling. In terms of pigments biosynthesis, colorimetric assays are used as the most intuitive and effective high-throughput screening method. For production of high-purity lycopene, we have created a CrtYB variant with solely phytoene synthase activity via directed evolution, which eliminated the metabolic flux loss towards β-carotene and dramatically improved lycopene yield (Xie et al. 2015a). Apart from altering substrate specificity, the catalytic activity of the pathway enzymes such as OBKT (β-carotene ketolase) and GGPPS (geranylgeranyl diphosphate synthase) was also improved by directed evolution, leading to improved biosynthesis of astaxanthin (Zhou et al. 2017b).

With the growing number of structural data and increasing availability of mechanistic information, rational design is playing a more important role in protein engineering (Fig. 4II). In this approach, engineering targets can be identified via homologous modeling followed by molecular docking or molecular dynamics simulations or via sequence conservatism analysis, followed by site-specific mutagenesis, which significantly reduces the onerous task of screening that is required for directed evolution. For instances, isopentenyl phosphate kinase (IPK) can catalyze the conversion of dimethylallyl alcohol into the fundamental building block of isoprenoids, IPP and DMAPP. Rational design based on protein coevolution analysis generated a positive IPK mutant with eightfold activity improvement, leading to 97% higher β-carotene production (Liu et al. 2016b).

##### Cofactor engineering

Biosynthetic pathways of pigments commonly comprise multiple redox reactions driven by the reducing forces provided by cofactors, NAD(P)H. Although native cofactor systems exist in microorganisms and plants, they are often insufficient to support heterologous biosynthesis.

Regulation of endogenous cofactor systems, via strengthening cofactor generation or restraining cofactor consumption, provides a feasible strategy to maintain cellular redox balance (Fig. 4 III). In *S. cerevisiae*, enhancement of NADPH supply by overexpression of POS5 (a mitochondrial NADH kinase) and ZWF1 (glucose-6-phosphate dehydrogenase involved in the pentose phosphate pathway) led to 59.9% and 81.4% increase of lycopene and β-carotene production, respectively (Zhao et al. 2015). In addition to regulation of endogenous cofactor systems, NADPH generation can also be enhanced via introduction of heterologous cofactor regeneration systems (Fig. 4 III). By introducing a heterologous Entner–Doudoroff (ED) pathway from *Zymomonas mobilis* into *E. coli*, the NADPH regeneration rate was increased by up to 25-fold, achieving 97% improvement of carotenoid production (Ng et al. 2015). Comprehensive regulation of NADH supply in *Monascus purpureus* by addition of exogenous cofactor compounds and disturbing nuoI (encoding NADH-quinone oxidoreductase) significantly increased *Monascus* pigment production (Liu et al. 2021a).

##### Transporter engineering

In metabolic engineering, extracellular export of natural compounds like fat-soluble compounds (e.g., carotenoids) is a big challenge. In recent years, based on transcriptomic analysis and literature mining (Fig. 4 IV), many novel transporters have been revealed for specific pigments and then applied to eliminate the transport bottlenecks.

Depending on the mechanism, transporters can be divided into the ATP-binding cassette (ABC) transporters (driven by ATP hydrolysis) and facilitator superfamily (making use of electric gradients). When microorganisms or plants are exposed to noxious environment or engineered to produce heterologous biochemicals, efflux pumps will be activated to export harmful substances for cell survival. Based on this mechanism, the transcriptomes of two engineered *S. cerevisiae* strains with different carotenoids production capacities were comparatively analyzed, suggesting induction of genes involved in pleiotropic drug resistance including the ABC transporter-pdr10 in the strain with high carotenoids production (Verwaal et al. 2010). To confirm its effects on secretion of carotenoids, *pdr10* from *S. cerevisiae* was introduced into *Rhodosporidium toruloides* in our previous study, leading to production improvement of total carotenoids from 1.9 μg/mg to 2.9 μg/mg in a bi-phasic culture (Lee et al. 2016). In addition to omics analysis, literature retrieval has also been used for transporter mining. For example, an array of efflux pumps reportedly responsible for secondary metabolite transports were introduced in anthocyanins-producing *E. coli*, and an anthocyanin-associated transporter *yadH* was identified, overexpression of which resulted in 15% increase of anthocyanin production (Lim et al. 2015).

#### Engineering of the metabolic network

##### Push–pull–block strategy

In heterologous biosynthesis, the efficiency of cell factories is often limited by the insufficient precursor supply and strong competitive metabolic branches. To direct more metabolic flux to the target compound, “push–pull–block” strategies have been developed and employed for biosynthesis of diverse chemicals and biofuels (Li et al. 2016): “push” indicates pushing more precursor supply towards target products, “pull” means strengthening the target biosynthetic pathway, and “block” or “restrain” represents downregulation of the competing pathways (Fig. 5A).

Isoprene is the monomeric building block of carotenoids. In our previous study, a rational push–pull–block strategy was employed for enhancement of isoprene biosynthesis in *S. cerevisiae* via engineering of the native acetyl-CoA and MVA pathways. This strategy was decomposed into upregulation of precursor supply in the acetyl-CoA and MVA pathway (by overexpression of ACS2/ERG10 and tHMG1, respectively), increasing the isoprene branch flux (via overexpression of IDI1 and ISPS), and downregulation of the competitive pathway (through decreasing the promoter strength of ERG20). With this combined regulation strategy, a total of 782-fold improvement of isoprene production was achieved in the engineered strain (Lv et al. 2014). Similar strategies have also enhanced the synthesis of pigments like flavonoids (Lyu et al. 2019b).

##### Inter- and intra-modular pathway engineering

In heterologous biosynthesis of complex pigments, overexpression of a large set of pathway genes often results in severe imbalance in pathway flux. Multivariate modular metabolic engineering partitions the complicated pathway of natural compounds into multiple modules, and the carbon flux of each individual module will be maximized and balanced to enable global fine-tuning of the whole metabolic network. The metabolic levels of different modules can be adjusted via altering plasmid copy number, promoter strength, RBS variety and enzyme properties (Fig. 5B). For example, the eight-step pinocembrin biosynthetic pathway was divided into four modules and optimized via altering the copy number of plasmids, leading to more than tenfold increase in pinocembrin production (Wu et al. 2013).

In addition to inter-module engineering, intra-module engineering is also viral to reduce the accumulation of intermediates (Fig. 5B). As illustrated in our previous study (Lv et al. 2016), a combinatorial strategy of protein engineering and modular pathway engineering was applied to simultaneously improve the intra- and inter-pathway balance for biosynthesis of isoprene in *E. coli*. Specifically, the whole biosynthetic pathway of isoprene was partitioned into the upstream module consisting of the native MEP pathway, and the downstream module containing isoprene synthase. The intra-module engineering within the upstream module via directed coevolution of DXS/DXR/IDI resulted in 60% improvement of isoprene production. Inter-module engineering between the upstream and downstream module via promoter manipulation further increased isoprene production by 4.7-fold. In a more recent study, a multidimensional heuristic process (MHP) was developed to co-ordinate intra-module activities and inter-module balance for biosynthesis of astaxanthin. A total of 15 genes were distributed in four modules and balanced by varying promoter strength, and intra-module balance was coordinated via RBS and enzyme variants, resulting in 320 mg/L of astaxanthin (Zhang et al. 2018a).

##### Multiplex genome engineering

Due to the complexity of biological systems, it is often hard to meet industrial demand on titers of desired compounds by solely engineering the target pathways. To date, a series of multiplex genome engineering methods have been developed for large-scale programming of cell factories, which can be classified into three types: (1) RNA interference-based (RNAi) method, for which a large RNAi library was coupled with high-throughput screening to generate accumulated beneficial modifications (Si et al. 2015). (2) Recombinase-based method, e.g., multiplex automated genome engineering (MAGE) (Wang et al. 2009) and trackable multiplex recombineering (TRAR) (Warner et al. 2010). (3) Multiplex CRISPR-based technologies, in which numerous gRNA or Cas enzymes are expressed simultaneously (McCarty et al. 2020). Herein, we will take MAGE (Fig. 5C) as an example to briefly introduce its application in metabolic engineering of *E. coli* for pigment production (Wang et al. 2009). MAGE depends on single-stranded DNA (ssDNA)-based genetic modification. Directed by λ-Red ssDNA-binding protein β, the sodas are loaded to the lagging strand of the replication fork for subsequent incorporation. Transformation of synthetic oligo pools enable generation of a diverse set of genetic modifications. Targeting 24 targeting genomic sites related to the 1-deoxy-D-xylulose-5-phosphate (DXP) biosynthesis pathway, fivefold increase of lycopene production was obtained with employment of MAGE method in *E. coli*.

#### Engineering of cellular systems

##### Dynamic control for cell growth–metabolism balance

Static pathway engineering, e.g., constitutive overexpression of pathway genes and deletion of competing pathways, has been proven as a powerful strategy to improve the yield of valuable products. However, it often results in decreased biomass due to severe metabolic burden and accumulation of toxic intermediates.

Two-phase cultivation has been widely recognized as a powerful strategy to circumvent the trade-offs between product accumulation and cell growth. Unlike traditional inducible expression systems, the newly developed environmental signal-responsive systems use cultivation parameters (like temperature, light, pH and oxygen) as inducers to trigger the switch-on and switch-off of gene expression, eliminating the requirement of adding expensive inducers (like isopropyl-β-D-thiogalactoside and galactose) (Fig. 6A). In our previous study, a glucose-responsive dynamic control system with P_*GAL*_ promoters has been developed by deletion of *GAL80*, which has been successfully applied for β-carotene production (Xie et al. 2014). By employment of a lycopene-indicated high-throughput screening method, a temperature-sensitive Gal4 mutant was screened out from directed evolution library and introduced into the *GAL80*-knockout yeast strain. With this design, target genes under P_*GAL*_ can be precisely switched on by altering the culture temperature, which resulted in 44% higher biomass and 177% increased lycopene production (Zhou et al. 2018), and facilitated high-density fermentation of astaxanthin (Zhou et al. 2019).

A further extension of dynamic control is autonomous regulation systems, in which target enzymes can be activated by intracellular intermediates or intercellular signals (Fig. 6A). For example, quorum-sensing circuits offer a powerful strategy to autonomously regulate cell metabolism. At high cell densities, quorum-sensing microbes can secrete sufficient molecular signals to alter the expression of endogenous genes. LuxI-LuxR and AHL-EsaR are two of the best-studied QS systems. A bifunctional QS circuit combining both LuxR and EsaR systems was developed using 3-oxohexanoyl homoserine lactone as the signal to dynamically regulate the expression of genes under P_*lux*_ and P_*esaR*_ in a cell density-dependent manner, and successfully improved flavonoid titer (Dinh and Prather 2019).

##### Co-culture engineering

Despite the progress in engineering single strains, introduction and regulation of the whole target biosynthetic pathway often raises overwhelming metabolic burden and encounter the intrinsic limitation of monocellular environment. Recently, co-culture engineering has emerged as an alternative approach for pathway engineering via division of the complex and long biosynthetic pathways into different host strains. As compared to mono-culture engineering, co-culture systems display notable advantages: (1) reducing metabolic burden by division of labor to different strains; (2) improving flexibility for balancing pathway modules via optimizing the population ratio of engineered strains; (3) providing diversified intracellular environments for functional overexpression of a large variety of enzymes involved; (4) reducing negative cross-interference among pathway modules; (5) enabling utilization of different carbon sources (Fig. 6B).

In recent years, considerable progress has been achieved in employment of modular co-culture system for biosynthesis of complex natural colored compounds (e.g., flavonoids and curcuminoids). One typical example is the biosynthesis of anthocyanins by *E. coli* polycultures, in which 15 exogenous enzymes were divided into 4 modules across four *E. coli* strains (Jones et al. 2017). Similar *E. coli* co-culture systems have also been adopted for biosynthesis of other flavonoids like sakuranetin (Wang et al. 2020), naringenin (Ganesan et al. 2017), resveratrol (Hong et al. 2020), and flavan-3-ols (Jones et al. 2016). In addition, efforts have also been made to employ cross-species co-culture systems for biosynthesis of complex compounds. For example, a synergistic *E. coli*-*S. cerevisiae* culture system was developed for naringenin production from D-xylose, generating 21.16 mg/L of the target product (Zhang et al. 2017). Although co-culture engineering exhibited significant advantages over mono-culture engineering, it is not suitable for all biosynthetic systems. For instance, many pathway intermediates cannot easily traverse cellular membranes, and different specifies may not be compatible in the same environment due to differing requirements of nutrients, temperature and pH.

## Major natural pigments and advances in biotechnological production

Depending on their structural characteristics, natural pigments may be classified into five major classes: tetrapyrroles, carotenoids, flavonoids, curcuminoids, and betalains. The general introduction of each class of pigments, including structure, classification, function and biosynthetic pathway, as well as the advances in biotechnological production of the typical products from each group are individually summarized in this section.

### Tetrapyrroles

#### Structure, classification and function

Tetrapyrroles, also called ‘pigments of life’, represent a small group of complex pigments which are the most abundant in living organisms. Structurally, tetrapyrroles are composed of four pyrrole-derived rings, joined together by methine bridges to form linear bile pigments or cyclic porphyrins (Solymosi and Mysliwa-Kurdziel 2017). Cyclic tetrapyrroles may differ in the oxidation state of pyrrole rings, peripheral substitutions, and the centrally chelated metal ions. They can be further subdivided into several groups—chlorophylls, hemes, cytochrome C, vitamin B_12_, and coenzyme F_430_, all of which contribute to the wide color range of natural pigments, from red, yellow to green, blue, and purple (Velisek et al. 2007). Moreover, degradation of cyclic tetrapyrroles generates linear tetrapyrroles like bilins, which loses one bridge carbon.

The most famous representatives of these ‘pigments of life’ are chlorophyll (‘plant blood’, in charge of the green color in plants) and heme (giving red color in animal blood). In natural producers, tetrapyrroles play important roles in a series of key metabolic processes, such as photosynthesis (e.g., chlorophyll) and electron transfer (e.g., cytochrome c) in plants, as well as transportation of oxygen (e.g., heme) in animals. More importantly, they have been proven to possess good antioxidant, anticancer, antimutagenic and anticlastogenic activities, showing great potential for applications in food and pharmaceutical industries (Beata and Solymosi 2016).

#### Biosynthetic pathway

**Fig. 7:**
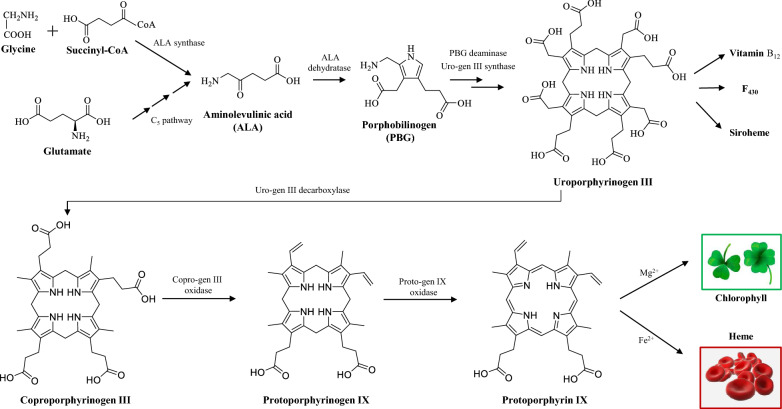
Schematic for the biosynthetic pathways of tetrapyrroles. Tetrapyrroles, including vitamin B_12_, F_430_, siroheme, chlorophyll and heme, are initiated from condensation of glycine and succinyl-CoA or synthesized through C_5_ pathway from the C_5_-skeleton of glutamate

The biosynthetic pathway of tetrapyrroles can be sectioned into six stages (Kobayashi and Masuda 2016) (Fig. 7): (1) biosynthesis of the common precursor- 5-aminolevulinic acid (ALA); (2) formation of the pyrrole unit porphobilinogen (PBG); (3) formation of uroporphyrinogen III, the carbon skeleton of porphyrins; (4) decarboxylation of uroporphyrinogen III to generate coproporphyrinogen III via uroporphyrinogen decarboxylase; (5) conversion of coproporphyrinogen III to protoporphyrinogen IX under the catalysis of coproporphyrinogen oxidase; (6) generation of protoporphyrin IX, the completely conjugated ring system to show color, by losing six hydrogen atoms under the action of protoporphyrinogen oxidase.

#### Typical product—heme

Heme is the most famous representative of tetrapyrroles, which gives red color in animal blood and serves critical biological functions in transporting O_2_ and electrons, modulating gene expression, as well as regulating protein stability and cell differentiation. Due to its natural red color displayed in blood, it has a long history of use as a meat additive. Nowadays, it is widely used as an iron supplement in the healthcare industry, owing to its high bioavailability (Hoppe et al. 2013), and is drawing increasing attention in production of plant-based meat alternatives by increasing the meat-like color and flavor (Gerhard 2020; Simsa et al. 2019).

Free heme sources are traditionally isolated from biological samples, typically animal blood, with obvious issues of this production mode being low-yielding and ethical animal welfare concerns. Bioproduction of heme on a large scale is therefore greatly preferred. The biosynthetic pathway of heme starts with the formation of 5-aminolevulinate (ALA) via the C_4_ pathway (in humans, animals, fungi and few bacteria) and the C_5_ pathway (in plant and most bacteria), followed by cyclization, modification of the side chains, and incorporation of reduced iron. In 2003, an entire heme biosynthetic pathway including *hemA* (encoding ALA synthase of the C4 pathway) from *Rhodobacter capsulatus*, *hemB*, *hemC*, *hemD* and *hemF* genes from *E. coli*, *hemE* from *Synechocystis*, and *hemY*, *hemH* from *B. subtilis* was assembled in *E. coli*, leading to accumulation of 3.3 μmol/L of heme (Kwon et al. 2003). In another study, co-expression of ALA synthase (hemA), NADP-dependent malic enzyme (MaeB) and dicarboxylic acid transporter (DctA) in *E. coli* resulted in 6.4 mg/L of heme (Kwon et al. 2009), and additional overexpression of pantothenate kinase (coaA) led to 0.49 μmol/g DCW of heme (Lee et al. 2013). In a more recent study, comprehensive regulation of heme synthesis and secretion was conducted to improve heme production. Respective expression of the C_4_ and C_5_ pathways in *E. coli* revealed the superior capacity of the C_5_ pathway for producing ALA. Downstream pathways for heme biosynthesis were subsequently optimized by elimination of the limiting steps and balancing enzyme expression. In addition, three competing pathways including acetate and lactate synthesis, and heme degradation pathway, were blocked via knocking out *pta* (encoding phosphate acetyl transferase), *ldhA* (encoding lactate dehydrogenase) and *yfeX* (encoding a heme dechelatase). The resulting *E. coli* strain produced 7.88 mg/L of heme. Further overexpression of a heme exporter cmABC led to total heme production of 239.2 mg/L, among which 151.4 mg/L was secreted (Zhao et al. 2018). These studies demonstrated that microbial metabolic engineering by enhancement of the upstream C4/C5 pathway, together with downregulation with the heme degradation pathway and improvement of heme secretion, provides a highly promising option for enhancing the production of free heme.

### Carotenoids

#### Structure, classification and function

Carotenoids are a family of lipophilic isoprenoid pigments produced by numerous bacteria (e.g., *Corynebacterium michiganense*, *Micrococcus roseus*, *Brevibacterium spp*., *Bradyrhizobium spp*., *Gordonia jacobaea* and *Dietzia natronolimnaea*), fungi (e.g., *Blakeslea trispora*, *Phycomyces blakesleeanus*, *Rhodotorula spp*., *Xanthophyllomyces dendrorhous*), and microalgae (e.g., the genus of *Chlorella*, *Dunaliella*, *Coelastrella an*d *Haematococcus*). (Carlos Mata-Gomez et al. 2014; Mannazzu et al. 2015; Mussagy et al. 2019a). Recent studies have demonstrated that some multicellular organisms like aphids, adelgids, phylloxerids, and gall midges, possess the capability for de novo synthesis of carotenoids (Cobbs et al. 2013; Moran and Jarvik 2010; Novakova and Moran 2012; Zhao and Nabity 2017); in contrast, most higher animals (including humans) can only take carotenoids from their diet (Rodriguez-Concepcion et al. 2018). Structurally, carotenoids originate from the condensation of C5 isoprenoid units to generate a vast class of over 600 carotenoid structures, among which C40 carotenoids are the most abundant in nature (Kiokias et al. 2016). These pigments are mainly classified into two subgroups: (1) hydrocarbons-carotenes (e.g., lycopene, α-carotene, and β-carotene); and (2) xanthophylls, the oxygenated derivatives of carotenes (e.g., lutein, zeaxanthin, astaxanthin, canthaxanthin). They display in yellow, orange, red or even colorless, and possess excellent antioxidant, anticancer, and anti-inflammation activities. According to the reports from Allied Market Research, the global carotenoid market value was $1.5 billion in 2017, and is estimated to reach nearly $2.1 billion by 2025 (Dawande 2018). Structurally, carotenoids originate from the condensation of C5 isoprenoid units to generate a vast class of over 600 carotenoid structures, among which C40 carotenoids are the most abundant in nature.

#### Biosynthetic pathway

**Fig. 8:**
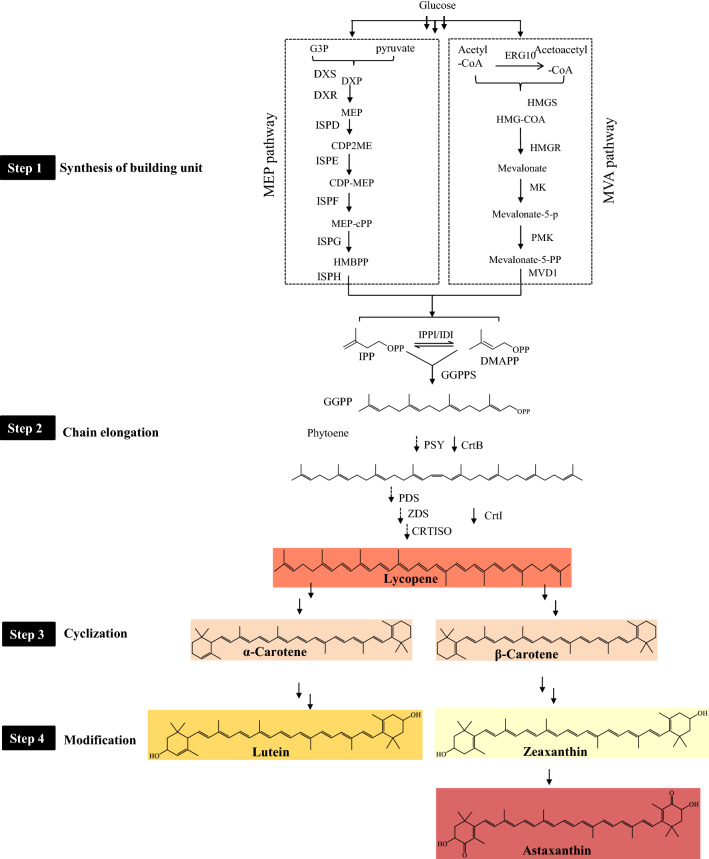
Schematic of carotenoids biosynthesis. Most carotenoids go through four synthetic steps consisting generation of building unit, chain elongation, cyclization, and modification. DXS, 1-deoxy-d-xylulose-5-phosphate synthase; DXR, 1-deoxy-d-xylulose-5-phosphate reductoisomerase; ISPD, 4-diphosphocytidyl-2C-methyl-D-erythritol synthase; IspE, 4-diphosphocytidyl-2-C-methyl-D-erythritol kinase; IspF, 2C-methyl-D-erythritol-2,4-cyclodiphosphate synthase; IspG, 1-hydroxy-2-methyl-2-(E)-butenyl-4-diphosphate synthase; IspH, 1-hydroxy-3-methyl-2-(E)-butenyl-4-diphosphate reductase; HMGS, HMG-CoA synthase; HMGR, HMG-CoA reductase; MK, mevalonate kinase; PMK, phosphomevalonate kinase; MVD1, mevalonate diphosphate decarboxylase; IPPI/IDI1, isopentenyl-diphosphate isomerase; GGPPS, GGPP synthase; PSY, phytoene synthase; PDS, phytoene desaturase; ZDS, ζ‐carotene desaturase; CRTISO, carotenoid isomerase; CrtB, phytoene synthase; CrtI, phytoene desaturase; DXP, 1-deoxy-D-xylulose-5-phosphate; MEP, 2-C-methyl-D-erythrito-l4-phosphate; CDP2ME, 4-diphosphocytidyl-2-C-methylerythritol; MEP-cPP, 2-C-methyl-D-erythritol-2,4-cyclo-diphosphate; HMBPP, (E)-4-hydroxy-3-methylbut-2-enyl-diphosphate; IPP, isopentenyl diphosphate; DMAPP, dimethylallyl diphosphate; GGPP, geranylgeranyl diphosphate

The biosynthetic process of carotenoids can be summarized as four steps, as shown in Fig. 8: (1) synthesis of the building units—isopentenyl diphosphate (IPP) and dimethylallyl diphosphate (DMAPP); (2) chain elongation by successive condensation reaction of IPP to DMAPP generating the growing polyprenyl diphosphate chain; (3)cyclization of linear isopentenyl pyrophosphate precursors to form the carotenoid carbon skeleton; (4) modification of the carotenoid carbon skeleton to generate diverse carotenoids. Among numerous carotenoids, β-Carotene and astaxanthin are the most commercially valuable products.

Scrutinizing the first step, IPP and DMAPP may generated from one of two distinct pathways, the mevalonate (MVA) pathway and the 2-C-methyl-D-erythritol 4-phosphate (MEP) pathway (Lange et al. 2000; Rohdich et al. 2003; Rohmer et al. 1993; Wölwer-Rieck et al. 2014). The MVA pathway is ubiquitous in bacteria, most fungi, and plants (in the cytoplasm), which initiates from acetyl-CoA and ends up with the production of DMAPP under the catalysis of acetoacetyl-CoA thiolase (ERG10), HMG synthase (HMGS), 3-hydroxy-3-methylglutaryl-CoA reductase (HMGR), MVA kinase (MK) and phosphor-MVA kinase (PMK), diphospho-MVA decarboxylase (MVD1), and IPP isomerase (IDI) (Allen et al. 1967; Vranova et al. 2013). In the MVA pathway, HMGR is identified as the rate-limiting enzyme. The MEP pathway is present in most bacteria, green algae, and plants (in the chloroplast) (Rohdich et al. 2003). It starts from the condensation of glyceraldehyde-3-phosphate (G3P) and pyruvate to generate 1-deoxy-D-xylulose 5-phosphate (DXP), which is further catalyzed by 1-deoxy-D-xylulose 5-phosphate synthase (DXS), DXP reductoisomerase (DXR), 2-C-methyl-D-erythritol 4-phosphate cytidylyltransferase (ISPD), 4-(cytidine 5′-diphospho)-2-C-methyl-D-erythritol kinase (ISPE), 2-C-methyl-D-erythritol 2,4-cyclodiphosphate synthase (ISPF), 4-hydroxy-3-methylbut-2-enyldiphosphate (HMBPP) synthase (ISPG), HMBPP reductase (ISPH), and IPP isomerase (IPPI), to yield IPP/DMAPP (the end product in the first step). The processes catalyzed by DXS (Lv et al. 2013a; Ramos et al. 2014; Xue and Ahring 2011), DXR (Lv et al. 2013a; Zhao et al. 2011) and IPPI (Lv et al. 2013b; Ramos et al. 2014) are identified to be the rate-limiting steps involved in the MEP pathway. In the second step, lycopene is the typical product from IPP/DMAPP, under catalysis of geranylgeranyl diphosphate synthase (GGPPS), phytoene synthase (PSY), phytoene desaturase (PDS), ZISO f-carotene isomerase (ZDS), and carotenoid isomerase CRTISO in plants, or under the catalysis of GGPP synthase (CRTE), phytoene synthase (CRTB), and phytoene desaturase/isomerase (CRTI) in non-photosynthetic bacteria. In the next step, α/β-carotene can be generated from lycopene under the catalysis of lycopene β-cyclase (LCYB) and/or lycopene ε-cyclase (LCYE). Further modification of carbon skeleton in the last step will result in other active carotenoids, e.g., lutein, zeaxanthin, and astaxanthin.

#### Typical product—β-carotene

β-Carotene is the most extensively studied carotenoid, abundant in leafy vegetables and is sold as a dietary supplement due to its well-known pro-vitamin A characteristic and excellent antioxidant activity. As the precursor of Vitamin A, β-carotene can help accelerate human growth, maintain a healthy immune system and good vision (Chen and Zhong 2015).

At present, the natural source-based production methods of β-carotene are either extraction from plants, silkworm excrement or algae, or cultivation of yeast and fungi. The extraction process of β-carotene from carrot, palm, seabuckthorn and other raw materials is complex. Moreover, its efficiency is limited by the long growth cycle of plants and the impact of climate and geography on their growth. Algae have proven to be more advantageous than plants in terms of productivity and production cycle. However, due to the harsh cultivation conditions and degradation issues, large-scale cultivation remains difficult. Microbial cultivation is a promising alternative with the advantage of continuous production, and has achieved stellar progress in the last decade (Table 1). *Blakeslea trispora* and *Rhodotorula glutinis* are the best-explored natural producers for β-carotene. The improvement in biosynthetic ability of these two strains have stemmed from the optimization of cultivation equipment^*133*^ and the culture medium (Chang-he et al. 2020; Tkáčová et al. 2017), random mutagenesis and addition of exogenous stimulants (Hu et al. 2013; Jing et al. 2016; Luo et al. 2021). The incomplete host genetic information and the lack of tools for genetic-level operation have thus far prevented more precise rational engineering of these strains. Researchers turned to engineering of *E. coli* and *S. cerevisiae*, the two model for prokaryotic and eukaryotic organisms, respectively. Herein we mainly focus on such studies reported in the last decade.

**Table 1:** Progresses of β-carotene biosynthesis in the last decade

Strain	Strategy	Yield	Year	Refs.
Native producers				
*Blakeslea trispora*	Development of new cultivation equipment	44.56 mg/g DCW, 8 days (bubble column reactor)	2012	Nanou et al. (2012)
*Blakeslea trispora*	Increase of oxygen concentration and induced high oxidative stress via addition of 3% (v/v) liquid paraffin	715 mg/L 84 h (shake flask)	2013	Hu et al. (2013)
*Mucor circinelloides*	Strain mutagenesis with UV and NTG	4 mg/g DCW (shake flask)	2016	Zhang et al. (2016b)
*Blakeslea trispora*	Addition of sodium acetate (NaAC) in mated *B. trispora*	59.91 mg/g DCW 2130 mg/L, 8 days (shake flask)	2016	Jing et al. (2016)
*Blakeslea trispora*	Optimization of cultivation by single factor and response surface test	523.8 mg/L (shake flask)	2020	Chang-he et al. (2020)
*Blakeslea trispora*	Protoplast fusion between ATCC 14,272( +) and ATCC 14,272(−)	36.93 mg/gDW (shake flask)	2021	Wang et al. (2021b)
*Blakeslea trispora*	Regulation of light and active oxygen	5.0 mg·g DW (shake flask)	2021	Luo et al. (2021)
*Rhodotorula glutinis*	Optimization of carbon:nitrogen (C/N) ratios (20:1, 50:1, 70:1, and 100:1) to analyze carotenoid and lipid biosynthesis	N.A	2017	Tkáčová et al. (2017)
*Rhodotorula glutinis*	Supplementation of vegetable oils as carbon source and optimization of bioreactor	0.36 mg/L 156 h (agitator bioreactor)	2019	Yen et al. (2019)
Heterologous hosts				
*S. cerevisiae*	Overexpressing HMG-CoA reductase gene and adding ergosterol synthesis inhibitor ketoconazole	6.29 mg/g DCW	2012	Yan et al. (2012)
*S. cerevisiae*	Deletion of gene *rox1*, *yjl064w* and *ypl062w*	~ 2.1 mg/g DCW	2013	Ozaydin et al. (2013)
*S. cerevisiae*	Design of a set of marker recyclable integrative plasmids (pMRI) for decentralized assembly of reconstructing controllable multi-gene pathways by employing the *GAL* regulatory system	7.41 mg/g DCW (shake flask culture)	2013	Xie et al. (2014)
*S. cerevisiae*	Introduction of *crtE*, *crtYB* and *crtI* from *Phaffia rhodozyma* into *S. cerevisiae* INVSc1; Additional expression of the catalytic domain of 3-hydroxy-3-methylglutaryl coenzyme A reductase (*cHMG1*)	528.8 ± 13.3 μg/g DCW (shake flask culture)	2014	Shi et al. (2014)
*S. cerevisiae*	Increasing the gene transcription of MVA pathway, by reducing temperature from 30℃ to 4℃;supplementation of 30 mg/L triclosan, an inhibitor of fatty acid synthesis	4.94 mg/g DCW (shake flask culture)	2015	Sun et al. (2015)
*S. cerevisiae*	Enriching unsaturated fatty acids (UFAs) contents by exogenous supplementation or strengthening their biosynthesis	2.83 mg/g DCW	2016	Sun et al. (2016)
*S. cerevisiae*	Discovery the relationship between carotenoid biosynthesis and cell membrane (CM) fluidity via genome-wide transcriptional analysis as well as optimization of CM fluidity by supplying linoleic acid	4.65 mg/g DCW (shake flask culture)	2016	Liu et al. (2016a)
*S. cerevisiae*	Development of an inducer/repressor-free sequential control strategy regulated by glucose concentration for two-phase cultivation of engineering strains	20.79 mg/g DCW (fed-batch fermentation)	2016	Xie et al. (2015a)
*S. cerevisiae*	Identification and overexpression of novel gene targets outside the isoprenoid pathway, including genes encoding 14–3-3 protein (Bmh1), class E protein of the vacuolar protein-sorting pathway (Did2), translation initiation factor (Tif5), and vacuolar H( +)-ATPase subunit 1 (Voa1)	5.9 ± 0.1 mg/g DCW	2017	Li et al. (2017)
*S. cerevisiae*	Introduction of a beta-carotene biosynthetic pathway containing crtYB, crtI, and crtE from *Xanthophyllomyces dendrorhous* in a xylose-fermenting *S. cerevisiae*	772.8 mg/L	2020	Sun et al. (2020)
*S. cerevisiae*	Expression of lipases of LIP2, LIP7, LIP8, and introduction of beta-carotene biosynthetic pathway from *Xanthophyllomyces dendrorhous*	46.5 mg/g DCW	2021	Fathi et al. (2021)
*E. coli*	Engineering MEP module and β-carotene synthesis module; engineering of ATP synthesis, pentose phosphate pathway (PPP) and TCA modules;	2.1 g/L 60 mg/g DCW (fed-batch fermentation)	2013	Zhao et al. (2013)
*E. coli*	Overexpression of the complete β-carotene synthetic pathway (including *dxs, ipiHP1, crtE, crtB, crtI,* and *crtY* genes) and the entire MVA pathway (namely, mvaE, mvaS, mvaK1, mvaK2, mvaD, and idi genes),	2.47 g/L 72 mg/g DCW (fed-batch culture)	2013	Nam et al. (2013)
*E. coli*	Improving the supply of precursor-IPP and GPP by optimization of MEP pathway and introduction of hybrid MVA pathway	256.8 mg/L in flask culture and 3.2 g/L in fed-batch fermentation	2014	Yang and Guo (2014)
*E. coli*	Engineering the cell membrane in both morphological and biosynthetic aspects by overexpressing membrane-bending proteins and engineering the membrane synthesis pathway	44.2 mg/g DCW (shake flask culture)	2017	Wu et al. (2017)
*E. coli*	Knockout of a few proteins related to the formation mechanism of outer membrane vesicles like *tolR* and *nlpI* were to promote the excretion of β-carotene; overexpression of both AccABCD and PlsBC to supplement the loss of membrane components	44.8 mg/g DCW (shake flask cultivation)	2019	Wu et al. (2019a)
*E. coli*	Regulation of central carbon metabolism by knockout of zwf and pts genes and improving NADPH supply by overexpression of nadk gene	266.4 mg/L in flask culture and 2579.1 mg/L in bioreactor fermentation	2020	Wu et al. (2020b)
*E. coli*	Integration of systems metabolic engineering, cell morphology engineering, inner- and outer-membrane vesicle formation, and cultivation optimization	343 mg/L	2021	Yang et al. (2021)
*Yarrowia lipolytica*	Optimization of promoter strength and gene copy number	4 g/L (fed-batch fermentation)	2017	Gao et al.( 2017)
*Yarrowia lipolytica*	Development of a combinatorial synthetic biology approach based on Golden Gate DNA assembly to screen the optimum promoter–gene pairs for each transcriptional step	6.5 g/L 90 mg/g DCW (fed-batch fermentation)	2018	Larroude et al. (2018)
*Yarrowia lipolytica*	Promoting the synthesis of precursor substrates by overexpression of hexokinase (Hxk) and hydroxymethylglutaryl-CoA synthase (Erg13)	2.4 g/L (fed-batch fermentation)	2020	Qiang et al. (2020)
*Yarrowia lipolytica*	Construction of codon-adapted CarRA and CarRB and maintaining metabolic balance by regulation of the expression level of enzymes involved in rate-limiting steps	1.7 g/L and 21.6 mg/g DCW (fed-batch fermentation)	2021	Liu et al. (2021b)

Via multiple strategies such as coordinating the inherent MEP pathway (Yuan et al. 2006) and β-carotene pathway (Zhao et al. 2013), introducing exogenous MVA pathway to increase the supply of precursors (Yang and Guo 2014; Yoon et al. 2009), cofactor engineering (Wu et al. 2020b), increasing membrane biosynthesis (Wu et al. 2017) and changing membrane morphology (Wu et al. 2019a) as well regulation of inner- and outer-membrane vesicle formation (Yang et al. 2021) to alleviate the burden caused by accumulation of hydrophobic macromolecule products on cell membrane, the β-carotene production of *E.coli* reached as high as 44.8 mg/g DCW. However, concerns such as the food safety of *E. coli*, the requirement of expensive inducers, and its susceptibility to phage infection, have thus far prevented the commercialization of β-carotene produced by engineered *E. coli*.

As a generally recognized as safe (GRAS) microorganism, *S. cerevisiae* has been widely used to produce carotenoids as well as other isoprenoids. In the early stage, the main research focuses were on improving the genetic stability of β-carotene synthetic genes in engineered strains and optimizing culture temperature (Shi et al. 2014; Sun et al. 2015). Overexpression of the rate-limiting enzyme in the MVA pathway and several novel genes outside the isoprenoid pathway and adjustment of the copy number of downstream pathway genes were then employed to balance the synthetic pathway and improve β-carotene production (Li et al. 2017; Yan et al. 2012). Deleting genes in bypassing pathways including lipid, amino acid and ergosterol syntheses, genes encoding transcription factors (e.g., Rox1) that represses many enzymes in the MVA and ergosterol synthesis pathways, as well as some genes involved in the stability of mRNA and protein (Ozaydin et al. 2013) also resulted in increased the accumulation of precursors and further increased final production of β-carotene. In addition, dynamic regulation of the synthetic pathway to separate β-carotene accumulation from cell growth greatly improved β-carotene production (Xie et al. 2015b). Recently, it was found that the synthesis and addition of unsaturated fatty acids also had a significant influence on β-carotene synthesis (Liu et al. 2016a; Sun et al. 2016).

The oleaginous yeast *Yarrowia lipolytica* has emerged as a promising microbial cell factory, due to its biochemical characteristics such as intrinsic high flux of acetyl-CoA and therefore a native high capacity to accumulate lipid-based chemicals. By strengthening and balancing the biosynthetic pathway (Gao et al. 2017; Larroude et al. 2018; Liu et al. 2021b; Qiang et al. 2020), production of up to 6.5 g/L β-carotene was achieved in *Y. lipolytica*.

#### Typical product—astaxanthin

Astaxanthin (3, 3′-dihydroxy-β,β-carotene-4,4′-dione), a red ketocarotenoid belonging to the terpene family, has received intensive attention worldwide due to its strong antioxidant activity and in particular, widespread use as feed additive for farmed salmon. The global market for astaxanthin was valued at USD 1,371.24 million in 2020 and is estimated to increase at an annual growth rate of 16.8% by 2028 (https://www.grandviewresearch.com/industry-analysis/global-astaxanthin-market). In recent years, microbial biosynthesis has emerged as a promising alternative astaxanthin production route to alga extraction and chemical synthesis. As summarized in Table 2, heterologous production of astaxanthin in microorganisms has been achieved in *E. coli*, *S. cerevisiae*, *Y. lipolytica* and *Corynebacterium glutamicum *^*158*^.

**Table 2:** Engineering microorganisms for astaxanthin production

Strain	Strategy	Cultivation mode	Astaxanthin yield	Refs.
*E. coli*	Gene screening (four *CrtZ* and twelve *CrtW* genes) and gene combination	Shake flask	1.99 mg/g DCW	Scaife et al. (2012)
*E. coli*	Genomic integration (*crtE*, *crtB*, *crtI*, *crtY* and *crtZ* genes from *P. ananatis* as well as *crtW148* gene from *N. punctiforme* PCC 73102) and promoter engineering	Shake flask	1.4 mg/g DCW	Lemuth et al. (2011)
*E. coli*	Optimization of gene expression via ribosome-binding site combinatorics	Shake flask	5.8 mg/g DCW	Zelcbuch et al. (2013)
*E. coli*	Gene mining of *Crt*E and *Crt*Z genes from *Sphingomonas sp. ATCC 55669*	Shake flask	6.6 mg/g DCW	Ma et al. (2016)
*E. coli*	Combinatorial expression of different *β*-carotene ketolase and ketolases	Shake flask	7.4 ± 0.3 mg/g DCW	Lu et al. (2017)
*E. coli*	Pathway engineering- metabolic engineering of DXP pathway by introduction of genes from *Kocuria gwangalliensis*, as well as introduction of astaxanthin downstream biosynthetic pathway from *Paracoccus haeundaensis*	Shake flask	1100 μg/g DCW	Jeong et al. (2018)
*E. coli*	Optimization of gene codon, promoters, strain species and culture media	Shake flask	4.30 ± 0.23 mg/g DCW 24.16 ± 2.03 mg/L	Li and Huang (2018)
*E. coli*	Comprehensive metabolic engineering, consisting of optimization of β-carotene biosynthetic pathway, introduction of *Crt*Z from *Pantoea ananatis* and *Cr*BKT from *Chlamydomonas reinhardtii*, truncation of *Cr*BKT, culture condition optimization, strengthening of DXP pathway and uptake of glycerol, introduction of hok/sok system for improving the stability of hereditary stability	Fed-batch fermentation	432.82 mg/L	Park et al. (2018)
*E. coli*	Optimization of the localization of *Crt*Z and *Crt*W enzymes; a total of 215.4% improved production of astaxanthin was achieved by combining *Crt*Z and *Crt*W together with a linker and locating them on the cell membrane	Shake flask	No clear data	Ye et al. (2018)
*E. coli*	Multidimensional heuristic process was proposed to optimization of the long astaxanthin biosynthetic pathway, via inter-module balance by varying promoter strength and intra-module balance by using different RBSs	Shake flask	15.1 mg/g DCW 320 mg/L	Zhang et al. (2018b)
*E. coli*	Optimization of gene expression by using different inducible and constitutive promoters	Shake flask	8.3 mg/g DCW	Chou et al. (2019)
*E. coli*	Optimization of cell morphology and oxidative stress for increasing astaxanthin yield, via gene mining and gene deletion. A complementary temperature-sensitive plasmid was introduced to further balance cell growth and production accumulation	Fed-batch fermentation	432.82 mg/L	Lu and Liu (2019)
*E. coli*	Gene fusion of CrtW and CrtZ	Shake flask	576.4 μg/g DCW	Nogueira et al. (2019)
*E. coli*	Assembly of the key enzymes in the MVA pathway (ACAT, HMGS, HMGR) into multi-enzyme complexes via orthogonal protein reactions (SpyCatcher/SpyTag and SnoopCatcher/SnoopTag pairs)	Shake flask	1 mg/g DCW	Qu et al. (2019)
*E. coli*	Gene screening and enzyme fusion of CrtZ and CrtW, replacement of different linkers, carbon source optimization	Shake flask	26.16 mg/L (5.18 mg/g DCW)	Wu et al. (2019b)
*E. coli*	Coordinated expression of astaxanthin biosynthesis genes – CrtW, CrtZ, CrtY, and regulation of molecular chaperone genes groES-groEL in the beta-carotene producing strain CAR026 (3.6 g/L)	Fed-batch fermentation	1.18 g/L	Gong et al. (2020)
*C. glutamicum*	Balancing the metabolic flux of CrtZ and CrtW via selection and combination of different enzymes, optimization of RBS, and initiation codon	Shake flask	0.4 mg·L^−1^·h^−1^	Henke et al. (2016)
*C. glutamicum*	Gene fusion of CrtZ and CrtW (CrtZ-CrtW help to accumulation astaxanthin while CrtZ-CrtW cannot produce astaxanthin); usage of combined carbon sources—glucose and acetic acid	Shake flask	3.1 mg/g DCW	Henke and Wendisch (2019)
*C. utilis*	Expression of *CrtE, CrtB, CrtI, CrtY, CrtZ* and *CrtW* genes with constitutive promoters	Shake flask	0.4 mg/g DCW	Miura et al. (1998)
*Y. lipolytica*	Introduction of *CrtYB, CrtI,* and *CrtE* gene from *Phaffia rhodozyma*, overexpression of endogenous tHMG1, multi-copy integration of CrtZ and CrtW gene into the genome, deregulation of ERG9	96-well plates	3.5 mg/g DCW 54.6 mg/L	Kildegaard et al. (2017)
*Y. lipolytica*	Optimization of the synthetic pathway of β-carotene precursor; optimization of the copy number as well as gene origins of β-ketolase and β-hydroxylase	Fed-batch fermentation	285 ± 19 mg/L	Tramontin et al. (2019)
*K. marxianus*	Gene screening of CrtZ and integration of astaxanthin biosynthetic pathway	Shake flask	No clear information	Chang et al. (2015)
*K. marxianus*	Site mutation of CrtZ and overexpression of the key enzymes in the limiting steps	Batch fermentation	9.972 mg/g DCW	Lin et al. (2017)
*S. cerevisiae*	Overexpression of Crt genes from *Phaffia rhodozyma* or bacteria	Shake flask	29 μg/g DCW	Ukibe et al. (2009)
*S. cerevisiae*	Gene cloning, codon optimization and copy number optimization of CrtZ and BKT from *H. pluvialis*	Shake flask	4.7 mg/g DCW	Zhou et al. (2015a)
*S. cerevisiae*	Strengthening MVA pathway and β-carotene biosynthetic pathway, site-directed evolution of BKT, optimization of gene copy number, strain hybridization	Shake flask	8.10 mg/g DCW 47.18 mg/L	Zhou et al. (2017a)
*S. cerevisiae*	Combination of different CrtZ and CrtW genes from diverse origins, improvement of CrtZ promoter strength	Shake flask Fed-batch fermentation	4.5 mg/g DCW 81.0 mg/L	Chen et al. (2017a)
*S. cerevisiae*	Directed coevolution of β-carotene ketolase and hydroxylase, dynamic control of gene expression using temperature signal	Fed-batch fermentation	235 mg/L	Zhou et al. (2019)
*S. cerevisiae*	Introduction of CrtZ from *Agrobacterium aurantiacum*, atmospheric and room temperature plasma mutagenesis (ARTP)	Fed-batch fermentation	217.9 mg/L	Jin et al. (2018)
*S. cerevisiae*	Physical mutagenesis by ARTP and adaptive evolution driven by H_2_O_2_	Fed-batch fermentation	404.78 mg/L	Jiang et al. (2020)
*S. cerevisiae*	In vitro and in vivo recombination of diverse heterologous *CrtZ* and *CrtW* genes	Shake flask	6.05 mg/g DCW	Qi et al. (2020)

Pathway optimization of astaxanthin could be divided into two parts: the upstream pathway section converting acetyl-CoA to β-carotene and the downstream pathway section converting β-carotene to astaxanthin. The upstream pathway has often been engineered using common strategies for terpene biosynthesis, such as improving the metabolic flux of MVA or MEP pathway (Kildegaard et al. 2017; Park et al. 2018) and downregulating competitive pathways like ergosterol synthesis (Kildegaard et al. 2017). To maximize the conversion of β-carotene to astaxanthin, the substrate preference and activity of β-carotene hydroxylase and ketolase need to be balanced. The carotenoids profile and astaxanthin contents vary among different combinations of β-carotene hydroxylase and ketolase from different organisms (Lu et al. 2017; Qi et al. 2020). To balance the activity of these enzymes, strategies including RBS site combination (Zelcbuch et al. 2013; Zhang et al. 2018b), promoter engineering and copy number adjustment (Gong et al. 2020; Kildegaard et al. 2017; Tramontin et al. 2019) were used.

Protein engineering also contributes to improvement of astaxanthin biosynthesis, including directed evolution (Zhou et al. 2017a, 2019), fusion expression (Nogueira et al. 2019) and construction of multi-enzyme complex (Qu et al. 2019).

Except for the synthetic pathway and the enzymes thereof, the properties of the chassis cell also influence astaxanthin yield. As an excellent antioxidant, high oxidative pressure is supposed to increase astaxanthin content. Using hydrogen peroxide as selective pressure, ARTP (atmospheric and room temperature plasma) mutagenesis (Jiang et al. 2020) and CRISPRi method (Lu and Liu 2019) generated mutant strains with improved astaxanthin production, uncovering gene targets related to ROS level, chronological lifespan and cell morphology.

To sum up, the typical strategies for heterologous production of astaxanthin in microorganisms include: (1) enhancing the supply of β-carotene as the precursor; (2) balancing the activities of β-carotene hydroxylase and β-carotene ketolase by screening, protein engineering or expression level regulation; (3) uncovering new engineering targets by random mutagenesis and omics analyses.

### Flavonoids

#### Structure, classification and function

Flavonoids are a highly diverse group of plant secondary metabolites derived from the phenylpropanoid metabolisms. They are distinguished by the characteristic structural backbone of C6−C3−C6, consisting of two aryl rings linked by a heterocyclic ring. A variety of modifications across this backbone, e.g., hydroxylation, ring repositioning, acetylation, glycosylation, and methylations, result in the vast chemical diversity of flavonoids. Based on these structural modifications, flavonoids can be divided into six major categories: flavanones, flavones, isoflavones, flavonols, catechins, and anthocyanins (Dudnik et al. 2018).

Flavonoids are abundant in fruits, vegetables, grains, trees, and flowers, and possess a variety of biological functions. For example, flavonoids are responsible for the color of petals and buds as well as the aroma of flowers and fruits to attract pollinating insects. Specifically, most of flavanones, flavones, isoflavones, flavonols are light yellow or yellow-colored, whereas anthocyanin hues range from red, pink, magenta, to violet, purple, and blue. Flavonoids can also protect plants from diverse environmental stresses like UV, heat, frost, drought, flooding, etc., by modifying the flavonoid structure for survival (Panche et al. 2016). Apart from plants, many in vivo or in vitro assays also demonstrate potential health-promoting functions of flavonoids to humans. They have been recommended as a dietary functional ingredient because of their high antioxidant capacity and protective effects against many infectious and degenerative diseases, such as inflammation, cardiovascular diseases, cancers, Alzheimer’s disease (AD), and other age-related diseases (Carrizzo et al. 2013; Chirumbolo 2014; Rahman et al. 2006; Scalbert et al. 2005; Sekizawa et al. 2013; Xiao and Hogger 2015; Yamagata et al. 2015). These health-promoting bioactivities of flavonoids, together with their diverse colors and high solubility in water, broaden their usage in the field of pharmaceutics and make them excellent candidates of food colorants, functional food and beverages, and dietary supplements. The global flavonoid market is predicted to reach USD 1.06 billion by 2025. Notably, the increasing demand for anthocyanins as healthful coloring agents in the food and beverage industry is the major driving factor. As multiple anthocyanins have been approved by the European Food Safety Authority (Panel on Food and Nutrient Sources, 2013) for use in foods, their universal usage as alternatives to synthetic dyes is expected, once bioproduction capability meets demand.

##### Biosynthetic pathway

**Fig. 9:**
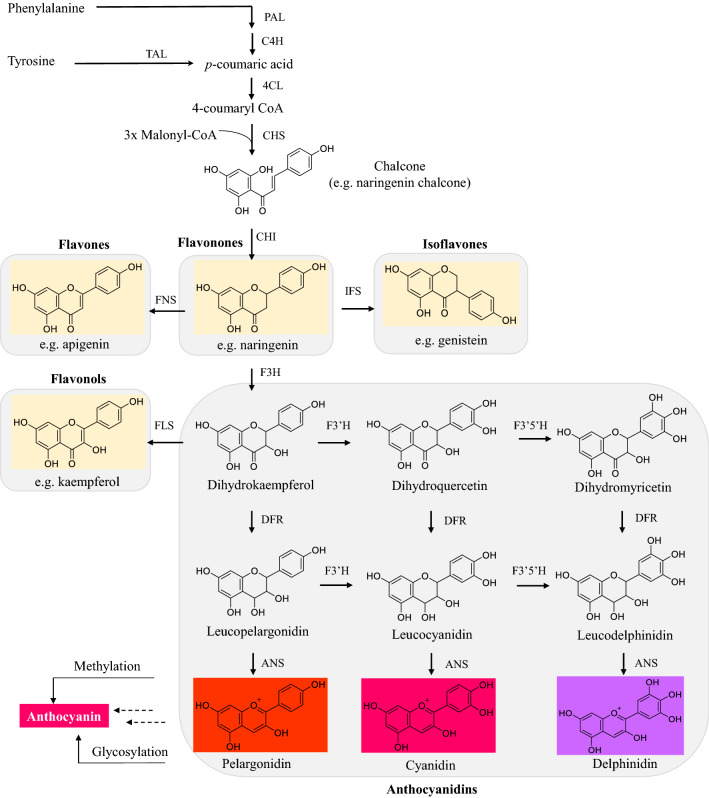
Schematic of flavonoids biosynthesis. PAL, phenylalanine ammonia lyase; C4H, cinnamate 4-hydroxylase; 4CL, p-coumaric acid: CoA ligase; TAL, tyrosine ammonia lyase; CHS, chalcone synthase; CHI, chalcone isomerase; FNS, flavone synthase; IFS, isoflavone synthase; F3H, flavanone 3-hydroxylase; FLS, flavonol synthase; F3′H, flavonoid 3′-hyroxylase; F3′5′H, flavonoid 3′,5′-hyroxylase; DFR, dihydroflavonol reductase; ANS, anthocyanidin synthase

As shown in Fig. 9 (Lyu et al. 2019a), flavonoids are derived from L-phenylalanine (Phe) or L-tyrosine (Tyr). Both amino acids can be converted into *p*-coumaric acid by non-oxidative deamination with distinct enzymes. Subsequently, *p*-coumaric acid: CoA ligase (4CL) mediates the addition of a coenzyme A group to *p*-coumaric acid to generate 4-coumaroyl-CoA, which was then condensed with three molecules of malonyl-CoA to create chalcones by chalcone synthase (CHS). Finally, chalcones are isomerized to flavanones through chalcone isomerase (CHI) or by spontaneous reactions, resulting in flavanone structure, e.g., naringenin. Naringenin can undergo various modifications by a series of “decorating” enzymes, giving rise to the vast diversity of flavonoids. For instance, the biosynthesis of flavanones or isoflavonones can be achieved by introduction of flavone synthase (FNS) or isoflavone synthase (IFS), respectively, whereas additional combination of flavanone 3-hydroxylase (F3H), dihydroflavonol reductase (DFR), anthocyanidin synthase (ANS) and anthocyanidin 3-O-glucosyltransferase (3GT) will lead to production of anthocyanins from flavanones.

#### Typical product—anthocyanins

Anthocyanins are water-soluble red, magenta and blue flavonoids. As multiple anthocyanins have already been approved by the European Food Safety Authority (Panel on Food and Nutrient Sources, 2013), their universal application as healthier and safer alternatives to synthetic dyes is expected imminently. Extensive genetic modification studies have been conducted to improve the productivity of anthocyanins in plants. Due to the complexity of the anthocyanin biosynthetic pathway, most strategies focused on regulation of R2R3 MYB transcription factors and MYC-like basic helix–loop–helix (bHLH) and WD40-repeat proteins. One typical example is enrichment of anthocyanins in tomato via expression of bHLH-type Delila (Del) and MYB-related factor Rosea1 (Ros1), which resulted in 2.83 mg/g (fresh weight) of anthocyanin (Butelli et al. 2008). As reviewed by Naing et al., great success has been achieved in metabolic engineering of plants (Naing and Kim 2018). However, this production approach as aforementioned, relies heavily on seasonal and environmental conditions and suffers from the difficulty of product purification as well as slow plant growth.

Microbial production provides an alternative approach to produce anthocyanin. In 2005, heterologous production of anthocyanins was first reported in *E. coli* (Yajun et al. 2005). A four-step metabolic pathway, consisting F3H from *Malus domestica*, DFR from *Anthurium andraeanum*, ANS from *M. domestica*, and 3-GT from *Petunia hybrid*, was constructed in *E. coli*, generating 5.6 μg/L of pelargonidin 3-O-glucoside and 6.0 μg/L of cyanidin 3-O-glucoside. In the following studies, systematic engineering strategies, including enhancing precursor supply (e.g., UDP-glucose), enzyme fusion, balancing gene expression level, and optimization of cultivation condition (e.g., pH), have been employed to further enhance the yield and titer of anthocyanin (Lim et al. 2015; Shrestha et al. 2019). As reported by Lim et al. (2015) and Shrestha et al. (2019), up to 350 mg/L and 439 mg/L of cyanidin 3-O-glucoside were obtained in *E. coli*, by using catechin as the substrate. Similar combinatorial engineering strategies also led to ~ 40 mg/L of cyanidin 3-O-glucoside in *Corynebacterium glutamicum* (Zha et al. 2018). More recently, de novo biosynthesis of anthocyanins has been made possible in *E. coli* and *S. cerevisiae*, via employment of a polyculture system and introduction of the whole pathway from phenylalanine to anthocyanin (Eichenberger et al. 2018; Jones et al. 2017). This approach greatly reduces cost via removing the need for addition of expensive flavonoid substrates during cultivation. Typical examples of metabolic engineering of plants and microorganisms for anthocyanins production are listed in Table 3.

**Table 3:** Typical examples for metabolic engineering of plants and microorganisms for enhanced production of anthocyanins

Target compound	Hosts	Strategies	Substrate	Yield	Year	References
Pelargonidin 3-O-glucoside (P3G) Cyanidin 3-O-glucoside (C3G)	*E. coli*	Introduction of F3H, DFR, ANS and 3GT	Naringenin, eriodictyol	5.6 μg/L, 6.0 μg/L	2005	Yajun et al. (2005)
PEG and C3G	*E. coli*	Gene screening, enhancement of UDP-glucose supply, culture medium pH adjustment, protein fusion	Afzelechin, catechin	78.9 mg/L of P3G, 70.7 mg/L of C3G	2007	Yan et al. (2008)
C3G	*E. coli*	Improvement of catechin uptake and C3G secretion; increase of ANS and 3GT expression; enhanced intracellular availability of UDP-glucose; optimization of culture and induction conditions	Catechin	350 mg/L	2015	Lim et al. (2015)
P3G	*E. coli*	Polycultures; 15 exogenous enzymes from diverse sources were divided into four *E. coli* strains	Glucose	9.5 mg/L	2017	Jones et al. (2017)
C3G	*E. coli*	Promoter engineering for balancing ANS and 3GT expression	Catechin	439 mg/L	2019	Shrestha et al. (2019)
Pyranoanthocyanins	*E. coli*	Co-culture of 4-vinylphenol or 4-vinylcatechol producing *E. coli* strains with cyanidin-3-O-glucoside producer recombinant *E. coli*	Catechin	19.5 mg/L of pyranocyanidin-3-O-glucoside-phenol, and 13 mg/L of pyranocyanidin-3-O-glucoside-catechol	2019	Akdemir et al. (2019)
Total anthocyanin	Tomato	Expression of the Delila (*Del*) and Rosea1 (*Ros1*) genes from the snapdragon *Antirrhinum majus*	/	2.83 mg/g (fresh weight)	2008	Butelli et al. (2008)
C3G	*Corynebacterium* *glutamicum*	Expression optimization of ANS and 3GT, improved UDP-glucose availability, process optimization	Catechin	40 mg/L	2018	Zha et al. (2018)
P3G, C3G, and delphinidin-3-O-glucoside (D3G)	*S. cerevisiae*	Gene screening, construction of the whole biosynthetic pathway of anthocyanins from glucose	Glucose	1–2 mg/L	2018	Eichenberger et al. (2018)
P3G	*S. cerevisiae*	Gene screening, genomic integration, deletion of EXG1 and SPR1, abolishing formation of phloretic acid, cultivation optimization	Glucose	0.001 µmol/g DCW	2018	Levisson et al. (2018)

### Curcuminoids—curcumin

#### Structure, classification and function

Curcuminoids are yellow polyphenolic compounds, with a typical structural backbone C6–C7–C6, derived from turmeric (the rhizome of the herb Curcuma longa mainly distributed in tropical and sub-tropical South-East Asia). As one of the most popular medicinal herbs, Curcuma longa has been used for the treatment of jaundice and other liver ailments in China, Japan, and India for many centuries. Curcumin is identified as the main active chemical constituent while bisdemethoxycurcumin and dicinnamoylmethane are found to be the other functional curcuminoids in turmeric. The studies on pharmacological activities of curcuminoids have been well reviewed previously (Amalraj et al. 2017), displaying prospects of curcuminoids in disease treatments. In addition to medical applications, curcuminoids have a long history as food coloring and flavoring agent. The World Health Organization (WHO) stated the acceptable daily intake of curcuminoids is in the range of 0–3 mg/kg. The safety of curcuminoids, as a food additive, has also been proved by FDA in USA (Amalraj et al. 2017).

#### Biosynthetic pathway

**Fig. 10:**
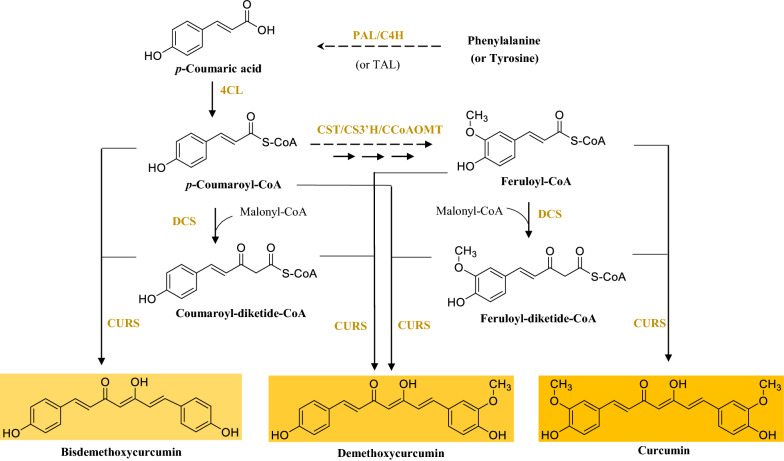
Schematic of curcuminoids biosynthesis. DCS, diketide-CoA synthase; CST, coumaroyl shikimate transferase; CS3′H, *p*-coumaroyl 5-O-shikimate 3′-hydroxylase; CCoAOMT, caffeoyl-CoA O-methyltransferase; CURS, curcumin synthase

Curcuminoids and flavonoids share the precursor pathway starting from phenylalanine or tyrosine to coumaroyl-CoA. For biosynthesis of curcuminoids (Fig. 10), coumaroyl-CoA will be converted to feruloyl-CoA first by the actions of *p*-coumaroyl shikimate transferase (CST), p-coumaroyl 5-O-shikimate 3’-hydroxylase (CS3’H), and caffeoyl-CoA O-methyltransferase (CCoAOMT). After that, curcuminoids can be produced by type III polyketide synthases, including diketide-CoA synthase (DCS) and curcuminoid synthase (CURS) (Lan et al. 2018).

#### Typical product—curcumin

Curcumin is the archetypal curcuminoid. It has been used as a natural coloring agent as well as food additive (to optimize food flavor) for centuries. For improved industrial commercialization, one effective strategy is to accelerate plant growth and induce plant defense systems via elicitation approaches. For example, bacterization of turmeric resulted in 13.6% increase of curcumin production (4.16 g/100 g) via promoting plant growth (Chauhan et al. 2017); elicitation by chitosan treatment improved the yield of curcumin by 100% (Sathiyabama et al. 2016). On the other hand, in vitro propagation provides another solution to resolve the problem of yield variability and low efficiency of vegetative propagation in traditional breeding. As reported by Pistelli et al., more than 260 μg/g fresh weight of curcumin was obtained from in vitro microrhizomes (Pistelli et al. 2012). In a more recent study, optimization of plant growth regulators, including sucrose, α-naphthalene acetic acid, 6-benzylaminopurine and light quality, achieved 534 μg/g of curcumin in in vitro microrhizomes of *Curcuma aromatic* (Wu et al. 2015).

Furthermore, in the past two decades, much efforts have also been made in the heterologous synthesis of curcumin in microorganisms (e.g., *E. coli* and *Aspergillus oryzae*) by synthetic biology (Table 4). By fine-tuning gene expression with employment of heat shock promoters (*dnaK* and *ibpA*) and optimization of RBS, 17 μM curcumin was obtained from ferulic acid in the strain expressing 4CL-DCS-CURS1 (Rodrigues et al. 2017). Optimization of cultivation conditions, including *E. coli* subspecies, induction parameters, culture media and carbon source concentration, led to high titers of curcumin (817.7 μM) from ferulic acid (Couto et al. 2017). In order to avoid addition of the expensive precursor ferulic acid, de novo biosynthetic pathway of curcumin from glucose or tyrosine was developed, via co-expression of TAL, 4-coumarate 3-hydroxylase (C3H), caffeic acid 3-O-methyltransferase (COMT), 4CL and CUS (or DCS and CURS1), while the yield of curcumin was quite low (0.2–0.7 mg/L) (Wang et al. 2015). In a later study, randomly modification of the 5’-untranslational region (UTR) sequences of all six enzymes involved in curcumin synthesis with design of MAGE oligomers and introduction of λ red recombination system resulted in approximately 38.2-fold improvement of curcumin production (3.8 mg/L) from glucose (Kang et al. 2018). In another study, in order to reduce cell metabolic burden caused by introduction of multiple genes, the whole curcumin biosynthetic pathway from tyrosine was divided into two modules and transferred into two separate *E. coli* strains, which led to 1.6-fold increase of final curcumin production (15.9 mg/L) (Rodrigues et al. 2020).

**Table 4:** Enhancement of curcumin production by metabolic engineering

Hosts	Strategies	Substrate	Yield	Year	References
*Curcuma longa*	In vitro propagation	/	260 μg/g fresh weight	2012	Pistelli et al. (2012)
*Curcuma aromatica*	In vitro propagation	/	534 μg/g	2015	Wu et al. (2015)
*Curcuma longa*	Chitosan elicitation	/	1.3 mg/g DCW	2016	Sathiyabama et al. (2016)
*Curcuma longa*	*Bacillus endophyticus* TSH42 and *Bacillus cereus* TSH77 were used for bacterization of rhizome in *C. longa*	/	4.16 g/100 g	2017	Chauhan et al. (2017)
*E. coli*	Introduction of 4CL, acetyl-CoA carboxylase (ACC) and CUS	Ferulic acid	57 mg/L	2008	Katsuyama et al. (2008)
*E. coli*	Co-expression of TAL, C3H, COMT, 4CL, DCS and CURS1	Tyrosine	0.2 mg/L	2015	Rodrigues et al. (2015)
*E. coli*	Co-expression of TAL, C3H, COMT, 4CL, and CUS	Tyrosine	0.67 mg/L	2015	Wang et al. (2015)
*E. coli*	Optimization of PBS; employment of heat shock promoters	Ferulic acid	17 μM	2017	Rodrigues et al. (2017)
*E. coli*	Optimization of cultivation conditions, including *E. coli* subspecies, induction parameters, culture media and carbon source concentration	Ferulic acid	817.7 μM	2017	Couto et al. (2017)
*E. coli*	Screening a library of 5’-UTR sequence mutants via MAGE	Glucose	3.8 mg/L	2018	Kang et al. (2018)
*Aspergillus oryzae*	Overexpression of CUS; Strengthening malonyl-CoA supply via disruption of *SnfA* and *SCAP*	Feruloyl-N-acetylcysteamine	404 μg/plate	2019	Kan et al. (2019)
*E. coli*	Gene expression optimization via replacement of plasmids; Co-culture system	Tyrosine	15.9 mg/L	2020	Rodrigues et al. (2020)
*E. coli*	Direction evolution of CUS, and cell membrane engineering via overexpression of monoglucosyldiacylglycerol synthase and supplementation of unsaturated fatty acid	0.6 g/L palmitoleic acid and 4 mM ferulic acid	1.46 mM curcumin	2020	Wu et al. (2020a)

Apart from *E. coli*, the filamentous fungus *Aspergillus oryzae* has also been used for production of curcuminoids via metabolic engineering. In a recent study, overexpression of CUS in *A. oryzae* was reported to produce 64 μg/plate of curcumin from feruloyl-N-acetylcysteamine (feruloyl-CoA analog). Enhancement of malonyl-CoA supply, via disruption of SnfA (an inhibitor of ACC enzyme) and SCAP (involved in acetyl-CoA-consuming sterol biosynthesis pathway), led to sixfold increase of curcumin yield (404 μg/plate) (Kan et al. 2019). From these studies, it can be understood that improving the de novo production ability and relieving the metabolic burden caused by introduction of multiple genes are key approaches for achieving high-yield production of curcumin by heterologous hosts.

### Betalains

#### Structure, classification and function

Betalains, also known as chromo-alkaloids, are water-soluble nitrogen-containing pigments. They are mainly present in plants of the order Caryophyllales and some higher fungi like *Amanita* and *Hygrocybe* genera. Betalamic acid is the common chromophore to all betalains. According to the structural differences in the betalain subgroups, betalains can be subdivided into betacyanins (red–violet) and betaxanthins (yellow–orange), whereby betacyanins are formed by conjugating betalamic acid with *cyclo*-dihydroxyphenylalanine (cDOPA) glucoside while betaxanthins result from the condensation of betalamic acid with amino acids or amines (Gandia-Herrero and Garcia-Carmona 2013). Typical betacyanins include betanin, isobetanin, probetanin, and neobetanin; and the main betaxanthins present in plants are vulgaxanthin, miraxanthin, portulaxanthin, and indicaxanthin.

In the plant kingdom, betalains and anthocyanins are uniquely mutually exclusive, having never been detected in the same plant despite their similar biological functions (mainly coloration). Different from anthocyanins, the color of betalains does not rely on pH (no change in the pH range of 3 to 7) and thus are good colorants for food with taste varying from sour to neutral. The main edible plants containing betalains include beets, Swiss chard, cactus fruit, and amaranth. Among them, red beetroot, the plant mostly cultivated in America and Britain, is the major commercially exploited source of betalains. Red beet pigments have been approved for usage in food processing by Europe (EU, E162) and the USA (FDA, Title 21 of Code of Federal Regulations-CFR-73.40) (Scotter 2011; Wrolstad and Culver 2012), commercialized as either liquid concentrates or spray-dried powders, containing from 0.3% to 1% of pigment, and are used as colorants for manufactured food products, such as red wine, meat, ice cream, soft drinks, sugar sweets, etc. (Akbar Hussain et al. 2018). They are also used as coloring agents in the pharmaceutical industry in drug formulations.

In recent years, betalains have also attracted increasing attention due to their pharmacological activities, including antioxidant, antimicrobial, and anticancer. Both fruit extracts rich in betalains and purified betalain compounds could act as free radical scavengers to protect proteins and NDA from oxidative damage (Escribano et al. 2017; Kumar et al. 2015; Sawicki and Wiczkowski 2018; Swarna et al. 2013). These discoveries would surely accelerate further development of betalains for application in the food and health care industries.

#### Biosynthetic pathway

**Fig. 11:**
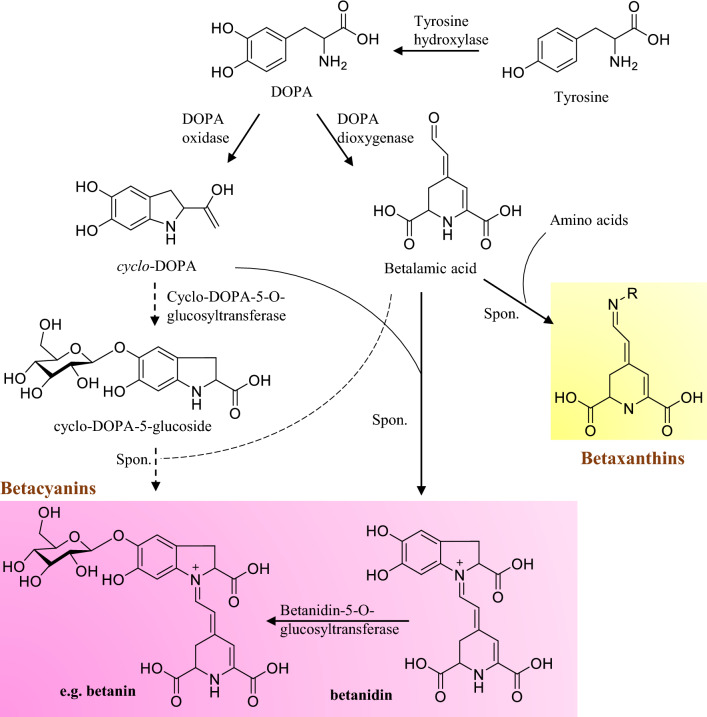
Schematic of betalains biosynthesis. Betalains can be subdivided into betacyanins (red–violet) and betaxanthins (yellow–orange), whereby betacyanins are formed by conjugating betalamic acid with cyclo-dihydroxyphenylalanine (cDOPA) glucoside while betaxanthins result from the condensation of betalamic acid with amino acids or amines. DOPA, dihydroxyphenylalanine

As compared with other important pigments like carotenoids and flavonoids, the biosynthetic pathway of betalains is much less well understood. By now, two pathways have been proposed (Gandia-Herrero and Garcia-Carmona 2013; Tanaka et al. 2008), of which the widely accepted one starts from tyrosine while the other, derived from tyramine, is still under exploration. The tyrosine pathway (Fig. 11) begins with the hydroxylation of tyrosine to dihydroxyphenylalanine (DOPA) under the catalysis of tyrosinase. Subsequently, DOPA may proceed in 2 divergent metabolic directions: either converted to betalamic acid by DOPA dioxygenase and spontaneous reaction, or to *cycol*-DOPA via a tyrosinase-catalyzed reaction followed by a spontaneous cyclization process.

#### Typical product—betanin

Among betalains, the main red pigment from red beet—betanin, is the most frequently utilized for commercial applications owing to its robust stability, high extinction coefficient and color intensity (Esatbeyoglu et al. 2015; Imtiyaj Khan and Giridhar 2015). In the food industry, betanin is commonly obtained from beet juice or root macerates via conventional solid–liquid extraction. Recent application of microwave-assisted extraction and high-pressure CO_2_-assisted extraction techniques (Cardoso-Ugarte et al. 2014; Ciriminna et al. 2018) have dramatically increased the extraction efficiencies. Besides, great efforts have been made in in vitro production of betanin via plant cell and tissue culture in diverse species (Georgiev et al. 2008), e.g., *Amaranthus tricolor* (Biswas et al. 2013), *Pereskia aculeata* Miller (Lage et al. 2015), and *Celosia cristata* (Warhade and Badere 2018). Selection of highly productive cell lines, optimization of inoculum conditions, addition of growth precursor, elicitation and permeabilization are also major effective strategies.

In parallel to the progress made in production enhancement from native producers, research has also been directed to heterologous biosynthesis of betalains via metabolic engineering. Identification of dihydroxyphenylalanine (DOPA) dioxygenase (DOD) enabled in vitro synthesis of yellow betaxanthins in *E. coli*, *Solanum tuberosum* (potato) and *Antirrhinum majus*, in the culture systems fed with the substrate DOPA (Gandia-Herrero and Garcia-Carmona 2012; Harris et al. 2012). Subsequently, with the discovery of new P450 enzyme *CYP76AD1* which is responsible for producing *cyclo*-DOPA from DOPA, heterologous production of betanidin (the precursor of betanin) in yeast was achieved by introduction of DODA1 from *Beta vulgaris* and *CYP76AD1* from beet (Hatlestad et al. 2012). In a recent study, CYP76AD1 is elucidated to catalyze both DOPA formation and subsequent conversion to cyclo-DOPA; and another P450 enzyme CYP76AD6 uniquely possesses tyrosine hydroxylase activity (Polturak et al. 2016). Based on discovery of enzymes in charge of the first committed step, de novo production of betalains has been successfully achieved in engineered tobacco, tomato, potato, eggplant, and ornamental petunia by expression of DODA1, CYP76AD1 and cycl0-DOPA-5-O-glucosyltransferase (cDOPA5GT) (Polturak et al. 2016, 2017). Similar operations in yeast led to 17 mg/L of betanin (Grewal et al. 2018) and two thirds of pigments were found to be secreted into the media, which would significantly reduce the efforts of pigment extraction as compared to plants. In a more recent study, expression of de-regulated arogenate dehydrogenase (ADH) resulted in sevenfold increase in betalain yield (855 mg/kg·FW) in planta (Timoneda et al. 2018). To date, these studies mainly focused on pathway mining and assembly, and more pathway optimization strategies are required to boost heterologous production of betalain.

## Challenges and future prospects

As compared with chemical synthesis, natural pigments produced using either natural producers or engineered organisms are currently not cost competitive, due to the generally low contents and high production cost. For example, the chemical synthesis of β-carotene was realized by using β-ionone as the precursor as early as 1960s, by Roche (since 1954) and BASF (since 1960). As compared with natural β-carotene derived from plants and cultivation of yeast, fungi, or algae, the price of chemical synthesis is two times cheaper. Nevertheless, according to the Global Market Size Analysis by GRAND VIEW RESEARCH, synthetic β-carotene sources only accounted for a very small share in the global industry due to the growing health awareness and various disadvantages regarding the usage of synthetic products (https://www.grandviewresearch.com/industry-analysis/beta-carotene-market). By contrast, natural β-carotene kept witnessing steady growth trend in these years, especially for algae route, which accounted for over 35% of total revenue in 2015. Therefore, utilization of natural pigments is the future trend in the relevant fields; however, how to reduce the production cost is one of the biggest issues to accelerate large-scale production and commercialization.

One main cost source for pigment biosynthesis is the cultivation expense. Specifically, strong light is required for cultivation of pigment-producing algae and the cell density is often limited. For plant extraction, there are additional issues of farmland occupation and climate dependence. Although microbial cultivation largely circumvents these issues, it currently suffers from the main bottleneck of high production costs especially when expensive glucose or sucrose is commonly used as the feedstock. To lower the cost of natural pigments, approaches should be developed for effective utilization of significantly cheaper agrowastes as the feedstocks, including proper pretreatment of biomass wastes and detoxification of the hydrolysates, as well as strain engineering towards efficient co-utilization of sugars and enhanced inhibitor tolerances.

Another obstacle for commercialization of natural pigments is the low productivity of natural producers. As mentioned before, great advances have been achieved by optimization of plant extraction, in vitro tissue culture, microbial cultivation, as well as heterologous synthesis by metabolic engineering to improve the productivity of bio-producers; whereas each approach still faces specific bottlenecks: (1) extraction from natural pigment-producing plants suffers from long cultivation cycle; (2) in vitro tissue culture encounters difficulties in scaling up; (3) pigment production by isolated microbial strains still encounters a glass ceiling because the regulatory mechanisms of all the different pigment biosynthesis are yet to be fully understood and microbial classis are often resistant to gene manipulations; moreover, some pigment-producing microorganisms may have biosafety issues; (4) using microorganisms with well-annotated genetic backgrounds (*E. coli*, *S. cerevisiae*, *B. subtilis*, *Y. lipolytica*, etc.) and sufficient manipulation tools as hosts to construct cell factories is therefore an emergent focus for biotechnological production of natural pigments. As compared with other high-valued compounds, the color characteristic of natural pigments endows heterologous engineering with huge advantages on protein engineering, directed-evolution, as well as sensor-based dynamic control, upon the natural high-throughput screening methods using pigments as direct indicators. Despite the significant advances made in biosynthesis of pigments via metabolic engineering, a number of challenges remain further down the road towards industrialization.

Challenge 1: Tricky enzyme expression issue. The yields of pigments produced by cultivation are generally low due to the incompatibility among the pathway enzymes and between the enzymes and the intracellular environment of the host. Dividing up a long biosynthetic pathway into multiple modules and matching them with compatible hosts after individual optimizations may thus serve as a more ideal approach for engineering complex pathways.

Challenge 2: Conflict between product accumulation and cell growth, caused by the accumulation of toxic intermediates and poor metabolic balance during static metabolic engineering. In order to allay this trade-off phenomenon, many strategies on dynamic regulation via two-stage cultivation and continuous control have been proposed. However, in many cases, the sensors and actuators for target compounds are unclear and the exploration is time-consuming. Besides, two-stage cultivation strategies will require sufficiently detailed mathematical models and improved genetic circuits (Venayak et al. 2015). These challenges offer the opportunity to motivate the integration of artificial intelligence, system biology and metabolic engineering.

Challenge 3: Lack of color diversity. Considering the rich pool of unculturable microorganisms, metagenomics may be conducted for mining of synthetic pathways towards novel pigments. Design of artificial synthetic pathways for generating brand-new pigments based on the understanding of the synthetic mechanism and chemical nature of existing pigments is another future direction.

Challenge 4: Storage of lipophilic pigments. Distinct with above three points, this challenge only occurs with heterologous production of lipophilic compounds, but not for hydrophilic products. The natural lipophilic nature of the pigments leads to their accumulation in cell membranes, which however exerts burden on the chassis cells. The generally higher production of carotenoids in oleaginous yeast and their storage in lipid droplets in natural algal producers implies storage of such products in natural or artificial subcellular compartments may be a promising solution.

Apart from above four challenges, safety is another major issue with the fermented pigments using genetically modified organisms for food application, with concerns about endotoxin and introduction of selective markers. The regulation (EC) No. 1829/2003 of the European Parliament and the Council made clear definition with the genetically modified food and feed sources, which are recognized to be the food products using genetically modified organisms (GMO) as the material, containing or produced from GMO. In EU, the commercialization of all GMOs and derived products like color additives must undergo risk assessment and regulatory approval by European Food Safety Authority (EFSA), according to the regulation (EC) No. 1829/2003. Detailed guidance for the risk assessment of genetically modified microorganism and their products as well as the presence at low level of genetically modified plant materials, are adopted by the EFSA GMO Panel in 2011 and 2017 (Naegeli et al. 2017; Organisms 2011). Based on the assessment of the taxonomic identity, safety concerns and the body of knowledge, many microorganisms belonging to filamentous fungi, bacteriophages, *Streptomycetes*, *Oomycetes*, *Enterococcus faecium* and *E. coli* were excluded in list of the qualified presumption of safety (QPS) of EFSA. This does not mean that all these strains are not allowed to be used the hosts to produce food products. As long as the safety of the strain is proven, it may still be authorized by the EU. Actually, to date, a number of natural products produced by genetic engineered *E. coli* has been authorized (Bampidis et al. 2020, 2019, 2021). If the genetic modification process does not introduce resistance genes, the possibility of passing the EFSA assessment will also be greatly improved. In future, more GRAS chassis systems should be explored with seamless cloning techniques for safe bioproduction of pigments.

## Conclusions

In summary, great strides have been devoted to the in vitro production of natural pigments via plant cell/tissue culture or optimization of microbial cultivation approaches. More remarkably, synthetic biology together with omics technologies have shed lights on the potential of development of heterologous systems for pigment production, by pathway exploration, pathway construction, and pathway optimization. Based on the studies in the last decade, omics technologies contributed significantly to mining of metabolic pathways and regulation mechanisms of pigment production; and substantial progress have been achieved by metabolic engineering strategies at the levels of parts, network and systems. With the advent and continuing rapid progress of novel biotechnologies, the prospect of large-scale commercial production of diverse natural pigments is likely to be realized in the near future.

## Data Availability

All data generated or analyzed during this study are included in this published article.
